# Supplementary material for: Generalist foundation models from a multimodal dataset for 3D computed tomography
Source: Nat Biomed Eng. 2026 Feb 12;10(8):1610–28. doi: 10.1038/s41551-025-01599-y (PMC13658014; doi:10.1038/s41551-025-01599-y)
Supplement: Supplementary file 1 [file 41551_2025_1599_MOESM1_ESM.pdf]

nature biomedical engineering

Supplementary information https://doi.org/10.1038/s41551-025-01599-y

Generalist foundation models from a multimodal dataset for 3D computed tomography

In the format provided by the authors and unedited

Table of Contents

Supplementary Table 1: Detailed overview of the CT-RATE dataset. ... 2
Supplementary Table 2: Finetuned text encoder's performance in automatic label extraction. ... 3
Supplementary Table 3: Hyperparameters for training each model of the multi-abnormality detection experiments. ... 3
Supplementary Table 4: Hyperparameters for (a) pretraining and (b) finetuning during each CT-CHAT ablation. ... 4
Supplementary Table 5: Statistical significance. ... 4
Supplementary Table 6: Predefined report generation questions. ... 5
Supplementary Table 7: The prompts that are used to generate conversation, description, free response, multiple choice, and text-only questions. ... 6
Supplementary Table 8: The prompt that is used to evaluate the clinical accuracy of the outputs generated by the VQA models using the LLM. ... 7
Supplementary Table 9: The system prompt for CT-CHAT. ... 7
Supplementary Table 10: Ablations of CT-CHAT for (a) image provision and (b) medical concept alignment. ... 8
Supplementary Table 11: Overview of anatomical segmentation in CT-RATE. ... 9
Supplementary Figure 1: Detailed ablation study. ... 10
Supplementary Figure 2: Illustration of confusion matrices for the fully supervised baseline model in the internal validation set. ... 11
Supplementary Figure 3: Illustration of confusion matrices for the CT-CLIP model in the internal validation set. ... 12
Supplementary Figure 4: Illustration of confusion matrices for the CT-CLIP model finetuned with VocabFine in the internal validation set. ... 13
Supplementary Figure 5: Illustration of confusion matrices for the CT-CLIP model finetuned with ClassFine in the internal validation set. ... 14
Supplementary Figure 6: Illustration of confusion matrices for the fully supervised baseline model in the RAD-ChestCT external validation set. ... 15
Supplementary Figure 7: Illustration of confusion matrices for the CT-CLIP model in the RAD-ChestCT external validation set. ... 16
Supplementary Figure 8: Illustration of confusion matrices for the CT-CLIP model finetuned with VocabFine in the RAD-ChestCT external validation set. ... 17
Supplementary Figure 9: Illustration of confusion matrices for the CT-CLIP model finetuned with ClassFine in the RAD-ChestCT external validation set. ... 18
Supplementary Figure 10: Clinical accuracy comparison of VQA models. ... 19
Supplementary Figure 11: Guardrail example to prevent hallucination of CT-CHAT when the CT volume is not provided. ... 20

a

Abnormality	Count in train set	Count in internal validation set	Train set ratio	Internal validation set ratio
Medical material	5818	313	0.123	0.103
Arterial wall calcification	13377	867	0.284	0.285
Cardiomegaly	5308	325	0.113	0.107
Pericardial effusion	3412	226	0.072	0.074
Coronary artery wall calcification	12025	765	0.255	0.252
Hiatal hernia	6751	417	0.143	0.137
Lymphadenopathy	12221	789	0.259	0.260
Emphysema	9122	600	0.193	0.197
Atelectasis	12263	713	0.260	0.235
Lung nodule	21382	1361	0.453	0.448
Lung opacity	17420	1184	0.369	0.390
Pulmonary fibrotic sequela	12589	831	0.267	0.273
Pleural effusion	5705	376	0.121	0.124
Mosaic attenuation pattern	3638	253	0.077	0.083
Peribronchial thickening	4973	355	0.105	0.117
Consolidation	8319	581	0.176	0.191
Bronchiectasis	4732	330	0.100	0.109
Interlobular septal thickening	3745	249	0.079	0.082

b

Manufacturer	Count in train set	Count in internal validation set	Train set ratio	Internal validation set ratio
Philips	28966	1928	0.614	0.634
Siemens	15946	971	0.338	0.320
PNMS	2237	140	0.048	0.046

c

Sex	Count in train set	Count in internal validation set	Train set ratio	Internal validation set ratio
Male	27462	1776	0.583	0.584
Female	19681	1263	0.417	0.416

Supplementary Table 1: Detailed overview of the CT-RATE dataset. a. The counts and proportions of various abnormalities identified within the training and validation subsets, offer insights into dataset composition. **b.** Details of the distribution of chest CT volumes according to manufacturer across both subsets, reflecting the dataset's diversity. **c.** The breakdown of chest CT volumes by sex in both subsets, provides a demographic perspective of the data.

Abnormality	Precision	Recall	F1 Score
Medical material	1.00	0.94	0.97
Arterial wall calcification	0.96	0.97	0.96
Cardiomegaly	1.00	0.96	0.98
Pericardial effusion	1.00	1.00	1.00
Coronary artery wall calcificatior	0.96	1.00	0.98
Hiatal hernia	0.96	0.96	0.96
Lymphadenopathy	0.94	1.00	0.97
Emphysema	1.00	0.96	0.98
Atelectasis	0.98	0.96	0.97
Lung nodule	0.95	1.00	0.98
Lung opacity	0.94	0.96	0.95
Pulmonary fibrotic sequela	0.97	0.96	0.96
Pleural effusion	1.00	1.00	1.00
Mosaic attenuation pattern	1.00	0.96	0.98
Peribronchial thickening	1.00	1.00	1.00
Consolidation	0.95	1.00	0.97
Bronchiectasis	1.00	0.91	0.95
Interlobular septal thickening	1.00	1.00	1.00

Supplementary Table 2: Finetuned text encoder's performance in automatic label extraction. This table presents a comprehensive assessment of performance metrics, including precision, recall, and F1 scores, for the finetuned text encoder across various abnormalities. The evaluation underscores the encoder's accuracy and efficiency in automating the extraction of labels for each abnormality.

	CT-Net (Baseline)	CT-CLIP (Zero-shot)	CT-CLIP (VocabFine)	CT-CLIP (ClassFine)
Optimizer	Adam	Adam	Adam	Adam
$\beta 1$	0.9	0.9	0.9	0.9
$\beta 2$	0.99	0.99	0.99	0.99
Learning rate	0.001	0.00000125	0.000	0.00001
Batch size	4	8	1	1
Schedular	N/A	N/A	Cosine annealing	Cosine annealing
Warmup steps	N/A	N/A	10,000	10,000
Max steps	N/A	N/A	20,000	20,000
GPUs utilized	1	4	1	1
Train duration	4 days	3 days	2 days	2 days
Iterations	100,000	100,000	20,000	20,000

Supplementary Table 3: Hyperparameters for training each model of the multi-abnormality detection experiments.

a

	CT-CHAT (Mistral 7B)	CT-CHAT (Vicuna 13B)	CT-CHAT (Llama 3.1 8B)	CT-CHAT (Llama 3.1 70B)
Automatic mixed precision	FP16	FP16	FP16	FP16
Per device batch size	12	12	12	12
Gradient accumulation steps	1	1	1	1
Learning rate scheduler	Cosine	Cosine	Cosine	Cosine
Warmup ratio	0.03	0.03	0.03	0.03
Peak learning rate	0.001	0.001	0.001	0.001
AdamW β	(0.9,0.999)	(0.9,0.999)	(0.9,0.999)	(0.9,0.999)
AdamW ϵ	1e-8	1e-8	1e-8	1e-8
Epochs	1	1	1	1

b

	CT-CHAT (Mistral 7B)	CT-CHAT (Vicuna 13B)	CT-CHAT (Llama 3.1 8B)	CT-CHAT (Llama 3.1 70B)
Automatic mixed precision	FP16	FP16	FP16	FP16
Per device batch size	3	3	3	3
Gradient accumulation steps	1	1	1	1
Learning rate scheduler	Cosine	Cosine	Cosine	Cosine
Warmup ratio	0.03	0.03	0.03	0.03
Peak learning rate	2e-5	2e-5	2e-5	2e-5
AdamW β	(0.9,0.999)	(0.9,0.999)	(0.9,0.999)	(0.9,0.999)
AdamW ϵ	1e-8	1e-8	1e-8	1e-8
Epochs	1	1	1	1

Supplementary Table 4: Hyperparameters for (a) pretraining and (b) finetuning during each CT-CHAT ablation.

Comparison	AUROC	F1 Score	Accuracy	Precision
CT-Net (Baseline) vs CT-CLIP (Zero-shot)	0.002	0.131	0.119	0.173
CT-Net (Baseline) vs CT-CLIP (VocabFine)	0.0002	0.016	0.006	0.049
CT-Net (Baseline) vs CT-CLIP (ClassFine)	0.0002	0.052	0.027	0.072
CT-CLIP (Zero-shot) vs CT-CLIP (VocabFine)	0.413	0.245	0.170	0.521
CT-CLIP (Zero-shot) vs CT-CLIP (ClassFine)	0.399	0.540	0.447	0.736
CT-CLIP (VocabFine) vs CT-CLIP (ClassFine)	0.998	0.599	0.544	0.775

Supplementary Table 5: Statistical significance. Using a two-sided unpaired permutation test with 10,000 permutations, we observe statistically significant differences ($p < 0.05$) in AUROC, F1 score, and accuracy between the supervised baseline model and each of the CT-CLIP models. For precision, significant differences are noted between the supervised baseline model and both finetuning.

Report generation questions

· Can you generate the report for the following chest CT image?
· Please provide the radiology report for the chest CT image mentioned.
· I need the radiology report for the given chest CT image.
· Could you create a report for this chest CT scan?
· Would you mind generating the radiology report for the specified chest CT image?
· Please generate the report for the chest CT image provided.
· Can you produce the radiology report for the attached chest CT image?
· I need a detailed report for the given chest CT image.
· Could you write the radiology report for this chest CT scan?
· Please give the radiology report for the specified chest CT image.
· Generate radiology report for the CT.
· Produce the report for this CT image.
· Write a radiology report for the following CT scan.
· Create a report for this chest CT.
· Provide the radiology report for this CT image.
· Can you generate the report for the following chest CT volume?
· Please provide the radiology report for the chest CT volume mentioned.
· I need the radiology report for the given chest CT volume.
· Could you create a report for this chest CT volume?
· Would you mind generating the radiology report for the specified chest CT volume?
· Please generate the report for the chest CT volume provided.
· Can you produce the radiology report for the attached chest CT volume?
· I need a detailed report for the given chest CT volume.
· Could you write the radiology report for this chest CT volume?
· Please give the radiology report for the specified chest CT volume.
· Generate radiology report for the CT volume.
· Produce the report for this CT volume.
· Write a radiology report for the following CT volume.
· Create a report for this chest CT volume.
· Provide the radiology report for this CT volume.
· Can you generate the report for the following chest CT scan?
· Please provide the radiology report for the chest CT scan mentioned.
· I need the radiology report for the given chest CT scan.
· Could you create a report for this chest CT scan?
· Would you mind generating the radiology report for the specified chest CT scan?
· Please generate the report for the chest CT scan provided.
· Can you produce the radiology report for the attached chest CT scan?
· I need a detailed report for the given chest CT scan.
· Could you write the radiology report for this chest CT scan?
· Please give the radiology report for the specified chest CT scan.
· Generate radiology report for the CT scan.
· Produce the report for this CT scan.
· Write a radiology report for the following CT scan.
· Create a report for this chest CT scan.
· Provide the radiology report for this CT scan.

Supplementary Table 6: Predefined report generation questions. These questions are randomly sampled while generating the report generation conversations.

Prompt to generate conversation questions

messages = [{"**role":** "system", **"content":** "You are a radiologist specializing in creating VQA (Visual Question Answering) datasets for Chest CT volumes. Generate three sets of conversation-style Q&A pairs based on the CT volume information, using the provided report as background without referring directly to it.

Enclose each set within tags <1>...<1>, <2>...<2>, and <3>...<3>. Use <q>...</q> for questions and <a>... for answers, ensuring most answers are derived from the images.

Example:
<1><q>Question 1</q><a>Answer 1<q>Question 2</q><a>Answer 2...<1><2><q>Question 1</q><a>Answer 1<q>Question 2</q><a>Answer 2...<2><3><q>Question 1</q><a>Answer 1<q>Question 2</q><a>Answer 2...<3>.

You can include as many question and answer couples as you find appropriate. Here is the report for you to get information about CT volume:\n"},

{"**role":** "user", **"content":** f"{report}"}]

Prompt to generate free response, description, and multiple choice questions

messages = [{"**role":** "system", **"content":** "You are a radiologist VQA dataset creator specializing in Chest CT reports. I need you to generate three sets of questions based on the given radiology report: free response, description, and multiple-choice questions. Each set should contain 3 Q/A pairs.

The free response questions should be enclosed within <free_response>...</free_response>. The description questions should be enclosed within <description>...</description>. The multiple-choice questions should be enclosed within <multiple_choice>...</multiple_choice>, with each question having 4 choices labeled as (a), (b), (c), and (d).

Ensure that the questions are specific to the Chest CT image described in the report.

Example for free response: <q>What abnormalities are present in the lung fields of this Chest CT image?<q>
Example for description: <q>Describe the findings shown in this Chest CT image.<q>
Example for multiple-choice: <q>Which of the following abnormalities are present in this Chest CT image?
(a) Choice 1 (b) Choice 2 (c) Choice 3 (d) Choice 4<q><a>Choice 2<a>

The structure should look like this:
<free_response><q>Question 1<q><a>Answer 1<a><q>Question 2<q><a>Answer 2<a><q>Question 3<q><a>Answer 3<a></free_response>
<description><q>Question 1<q><a>Answer 1<a><q>Question 2<q><a>Answer 2<a><q>Question 3<q><a>Answer 3<a></description>
<multiple_choice><q>Question 1 (a) Choice 1 (b) Choice 2 (c) Choice 3 (d) Choice 4<q><a>(b) Choice 2<a><q>Question 2 (a) Choice 1 (b) Choice 2 (c) Choice 3 (d) Choice 4<q><a>(a) Choice 1<a><q>Question 3 (a) Choice 1 (b) Choice 2 (c) Choice 3 (d) Choice 4<q><a>(c) Choice 3<a></multiple_choice>

Please always follow this format to create the questions and answers."},

{"**role":** "user", "**content**": f"{report}"}]

Prompt to generate text only questions

messages = [{"**role**": "system","**content**": "You are a chest CT dataset creator specializing in generating question-and-answer sets based on radiology articles focusing on chest CTs. I need you to create 5 question-and-answer pairs for each article.

Each question-and-answer pair should be enclosed within tags like <q>...<q> for questions and <a>...<a> for answers. Also include all output in <1>...<1> (remember to put one at the beginning one at the end).

For example, the structure should look like this: <1><q>Question 1<q><a>Answer 1<a><q>Question 2<q><a>Answer 2<a><q>Question 3<q><a>Answer 3<a><q>Question 4<q><a>Answer 4<a><q>Question 5<q><a>Answer 5<a><1>.

Please follow this format to create the conversation questions and answers. Make sure the questions are variable, educative, and the answers are long and instructive enough.The questions should be those that a student or radiologist might ask about chest CT images in general. Do not generate questions specific to the content or research idea of the provided article. Focus solely on general knowledge about chest CT imaging. Examples of such questions include:

- What are the typical imaging findings of interstitial lung disease on a chest CT?
- How is ground-glass opacity on a chest CT typically interpreted?
- What are the common causes of lymphadenopathy seen in chest CT scans?
- How can chest CT help in the diagnosis of pulmonary embolism?
- What are the distinguishing features of lung nodules on a chest CT?
- How are lung nodules found in chest CT scans managed?

The answers must be factually accurate and should be retrieved from the given article (but not about the case just the given factual knowledge). Do not reference specific details, cases, or research findings from the provided article. Ensure that the information in the answers is universally applicable to chest CT imaging. "},

{"**role**": "user", "**content**": f"{article_title}\n\n{article_content}"}]

Supplementary Table 7: The prompts that are used to generate conversation, description, free response, multiple choice, and text-only questions.

Prompt for clinical accuracy evaluation with Llama 3.1 70B

messages = [{"**role**": "system", "**content**": "**Role:** You are an expert medical evaluator specializing in the assessment of clinical accuracy for AI-generated reports and answers derived from 3D chest CT volumes. Your task is to compare the AI-generated answers with the provided ground truth answers and assign a clinical accuracy score based on specific evaluation criteria. Your assessment should focus on precision, relevance, and clinical soundness.

Scoring:

- **10/10:** The generated response is fully aligned with the ground truth—completely accurate, relevant, comprehensive, clear, and clinically sound.
- **7-9/10:** The generated response is mostly accurate, with minor discrepancies or omissions that do not significantly affect clinical interpretation.
- **4-6/10:** The generated response contains noticeable errors or omissions that could impact clinical understanding, though some correct information is present.
- **1-3/10:** The generated response has significant inaccuracies, irrelevant content, or lacks essential information, potentially leading to clinical misunderstanding.
- **0/10:** The generated response is completely incorrect or irrelevant, offering no clinical value.

Question Types: For each evaluation, you will be provided with the question type, question, ground truth answer, and generated answer.

- **Report Generation:** Requires a detailed narrative report summarizing findings from the 3D chest CT volume, covering all relevant clinical observations, including pathology, location, and significant findings. The report should follow the CT report format. Consider the formatting of the output for this question in addition to clinical accuracy. Non-existent pathologies are not necessarily required to be mentioned in the generated report. Do not deduct points if this is the case (e.g., "no pleural effusion" might not be in the output, but if "pleural effusion" is present, it must be included). Deduct points if the model hallucinates pathologies that are not in the ground truth.
- **Long Answer Questions:** Require comprehensive, detailed responses that thoroughly address the question, including necessary explanations or justifications.
- **Short Answer Questions:** Require brief, direct answers addressing the question with essential information, such as identifying pathology or confirming a diagnosis.
- **Multiple Choice Questions:** Require the selection of the most clinically accurate option from predefined choices, matching the ground truth answer. There should be only one correct answer for this question type. If more than one answer is provided, assign a very low score. If the answer is incorrect, assign a very low score as well.

Instructions: Please review the question type, question, ground truth answer, and generated answer. Assign a score out of 10 based on the evaluation criteria above. Provide only the score without additional commentary or explanation."},

{"**role**": "user", "**content**": f"

Question type: {question_type}

Question: {question}

Ground truth answer: {ground_truth}

Generated answer: {model_output}"}]

Supplementary Table 8: The prompt that is used to evaluate the clinical accuracy of the outputs generated by the VQA models using the LLM.

System prompt for CT-CHAT

messages = [{"**role**": "system", "**content**": " You are CT-CHAT, an AI assistant specializing in Chest CT imaging, dedicated to providing accurate and relevant information exclusively related to Chest CT scans and associated medical topics. You are equipped to answer questions and offer detailed analyses only when the CT volume/scan/image is provided, indicated by the <provided> token. If this token is not present and users inquire about specific findings, pathologies, or request descriptions related to a Chest CT, respond by requesting the necessary data with the phrase: "Please provide the CT volume." Once the <provided> token is present in the question, you are authorized to address questions about pathologies, anatomical or clinical findings, diagnostic descriptions, report generation, comparisons, or any other questions regarding the image. If it does not appear in the question, even when special tokens <multiple_choice>, <report_generation>, <long_answer>, and <short_answer> are given, ignore the question and ask for the CT volume. Always look for the <provided> token, even if there are special tokens. If there is a <provided> token in any question (including new and previous ones), never ask for the CT volume again and answer the question. You can ignore the <provided> token check and answer the question directly if and only if the question is about general medical knowledge, not about the provided CT volume (such as typical findings on a Chest CT or management of the patient). For example, "What are the typical imaging findings of acute respiratory distress syndrome (ARDS) on a chest CT?" is a general question not specific. If user asks a CT specific question after non-spesific question, look for the <provided> token as well even if the special tokens are given. It is crucial to avoid discussing topics outside of Chest CT imaging and directly related medical information, ensuring that all responses are clear, concise, and focused on the provided Chest CT data for the highest level of accuracy and relevance. If the user greets you with something like "hello," respond appropriately."},
{"**role**": "user", "**content**": f"{question}]}]

Supplementary Table 9: The system prompt for CT-CHAT.

a

Metric	Long answer		Short answer		Multiple choice		Report generation	
	without image	with image	without image	with image	without image	with image	without image	with image
↑ BLEU-1	0.383	0.482	0.248	0.274	0.827	0.838	0.125	0.395
↑ METEOR	0.259	0.295	0.140	0.155	0.568	0.577	0.085	0.218
↑ ROUGE-L	0.434	0.515	0.565	0.592	0.886	0.895	0.129	0.321
↑ CIDEr	2.234	3.126	1.715	1.821	7.776	7.853	0.003	0.220
↑ Llama Score	7.448	7.801	6.902	7.240	8.903	9.010	2.130	5.665

b

Metric	Long answer		Short answer		Multiple choice		Report generation	
	without pretrain	with pretrain	without pretrain	with pretrain	without pretrain	with pretrain	without pretrain	with pretrain
↑ BLEU-1	0.482	0.482	0.277	0.274	0.837	0.838	0.390	0.395
↑ METEOR	0.294	0.295	0.156	0.155	0.576	0.577	0.218	0.218
↑ ROUGE-L	0.514	0.515	0.596	0.592	0.893	0.895	0.323	0.321
↑ CIDEr	3.115	3.126	1.839	1.821	7.839	7.853	0.222	0.220
↑ Llama Score	7.816	7.801	7.271	7.240	9.009	9.010	5.785	5.665

Supplementary Table 10: Ablations of CT-CHAT for (a) image provision and (b) medical concept alignment. The concept alignment has minimal impact on model accuracy while providing the volumes is essential for the model to accurately answer questions, especially in report generation.

Segmentation counts for different structures

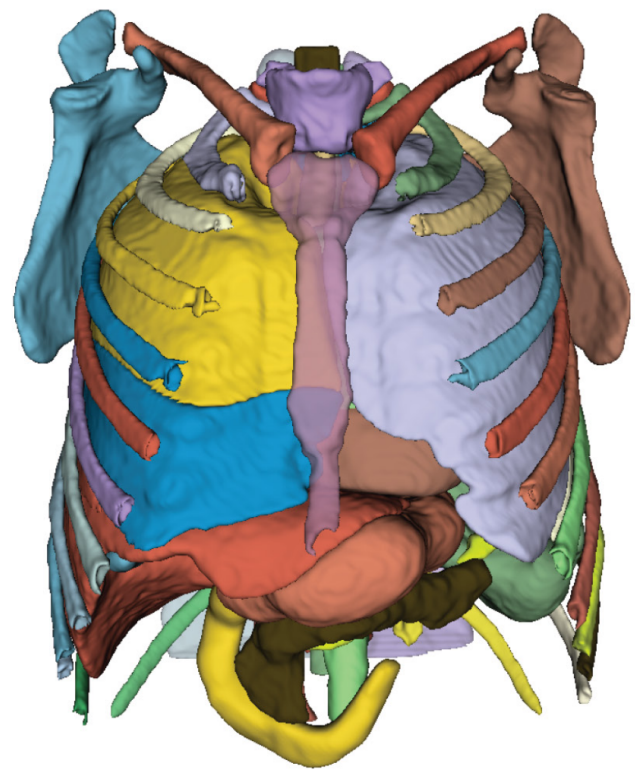

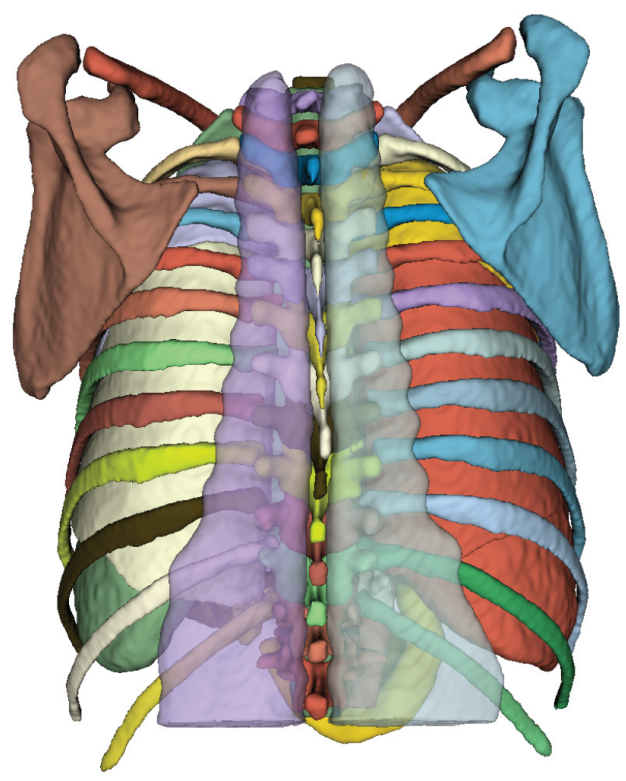

Cardiovascular system		
Structure name	Train count	Validation count
Heart	23864	1552
Myocardium	23855	1552
Heart atrium R/L	46037	2996
Heart ventricle R/L	45800	2973
Common iliac artery R/L	3091	256
Common iliac vein R/L	96	4
Pulmonary artery	23864	1551
Aorta	23877	1552
Inferior vena cava	23545	1526
Portal vein and splenic vein	22732	1463
Carotid artery R/L	43561	2775
Blood	272	12
Pericardium	23872	1552

Digestive system		
Structure name	Train count	Validation count
Liver	23867	1551
Gallbladder	18995	1213
Stomach	23397	1517
Pancreas	22967	1483
Duodenum	22288	1452
Small intestine	21042	1371
Colon	23041	1511
Sigmoid colon	761	40
Colostomy bag	23421	1526
Rectum	191	12
Oral cavity	3064	193
Esophagus	23861	1552
Cervical esophagus	21879	1425
Cricopharyngeus	17903	1170

Other structures		
Structure name	Train count	Validation count
Face	1034	50
Scalp	272	12
Buccal mucosa	853	46
Both lips	3661	262
Subcutaneous tissue	24128	1564
Prosthetic breast implant	2653	167

Muscular system		
Structure name	Train count	Validation count
Iliopsoas muscle R/L	26111	1628
Gluteus minius muscle R/L	55	2
Gluteus medius muscle R/L	110	6
Gluteus maximus muscle R/L	74	2
Deep muscle of back R/L	47764	3110
Psoas major muscle R/L	31527	2064
Rectus abdominis muscle R/L	47007	3066
Muscle of head	260	12
Muscle	24128	1564

Endocrine system		
Structure name	Train count	Validation count
Adrenal gland R/L	45260	2938
Thyroid	21918	1425
Gland structure	22682	1472
Pituitary gland	274	13

Reproductive system		
Structure name	Train count	Validation count
Prostate	27	1
Seminal vesicle	30	1
Mammary gland R/L	26164	1675

Urinary system		
Structure name	Train count	Validation count
Kidney R/L	43570	2823
Urinary bladder	60	5

Body cavities		
Structure name	Train count	Validation count
Thoracic cavity	24126	1564
Abdominal cavity	23901	1555
Mediastinum	23885	1553

Skeletal system		
Structure name	Train count	Validation count
Cervical vertebrae C1- C7	42659	2801
Thoracic vertebrae T1- T12	279608	18177
Lumbar vertebrae L1- L5	48860	3186
Right ribs 1-12	262026	17029
Left ribs 1-12	261880	17027
Sacrum	41	1
Hip R/L	162	11
Femur R/L	50	2
Clavicle R/L	43800	2847
Scapula R/L	47745	3105
Humerus R/L	44254	2884
Mandible	8105	534
Sternum	23870	1551
Spongy bone	272	12
Compact bone	272	12
Bones	24128	1564

Nervous system		
Structure name	Train count	Validation count
Brain	295	12
Brainstem	595	37
White matter	272	12
Gray matter	270	12
Cerebrospinal fluid	272	12
Spinal cord	24074	1562
Spinal canal	24096	1563
Optic chiasm	260	13
Optic nerve R/L	568	26

Respiratory system		
Structure name	Train count	Validation count
Upper lobe of lung R/L	47751	3105
Middle lobe of lung R	23866	1552
Lower lobe of lung R/L	47754	3104
Larynx	11840	730
Trachea	23873	1553
Arytenoid cartilage	9552	618
Glottis	14411	946
Supraglottis	6679	416

Sensory system		
Structure name	Train count	Validation count
Eyeball	267	12
Cochlear R/L	516	25
Anterior eyeball segment R/L	561	25
Posterior eyeball segment R/L	598	26

Immune system		
Structure name	Train count	Validation count
Spleen	23621	1536

Glandular system		
Structure name	Train count	Validation count
Parotid gland R/L	1151	65
Lacrimal gland R/L	572	24
Submandibular gland R/L	4290	271

Supplementary Table 11: Overview of anatomical segmentation in CT-RATE. The tables summarize the number of segmented anatomical structures across the training and validation splits. The accompanying illustration shows the segmented chest anatomical regions on a 3D CT scan, generated using a state-of-the-art segmentation model. These annotations are made publicly available in the CT-RATE repository to support downstream tasks such as anatomical localization, visual grounding, and future multi-abnormality segmentation research.

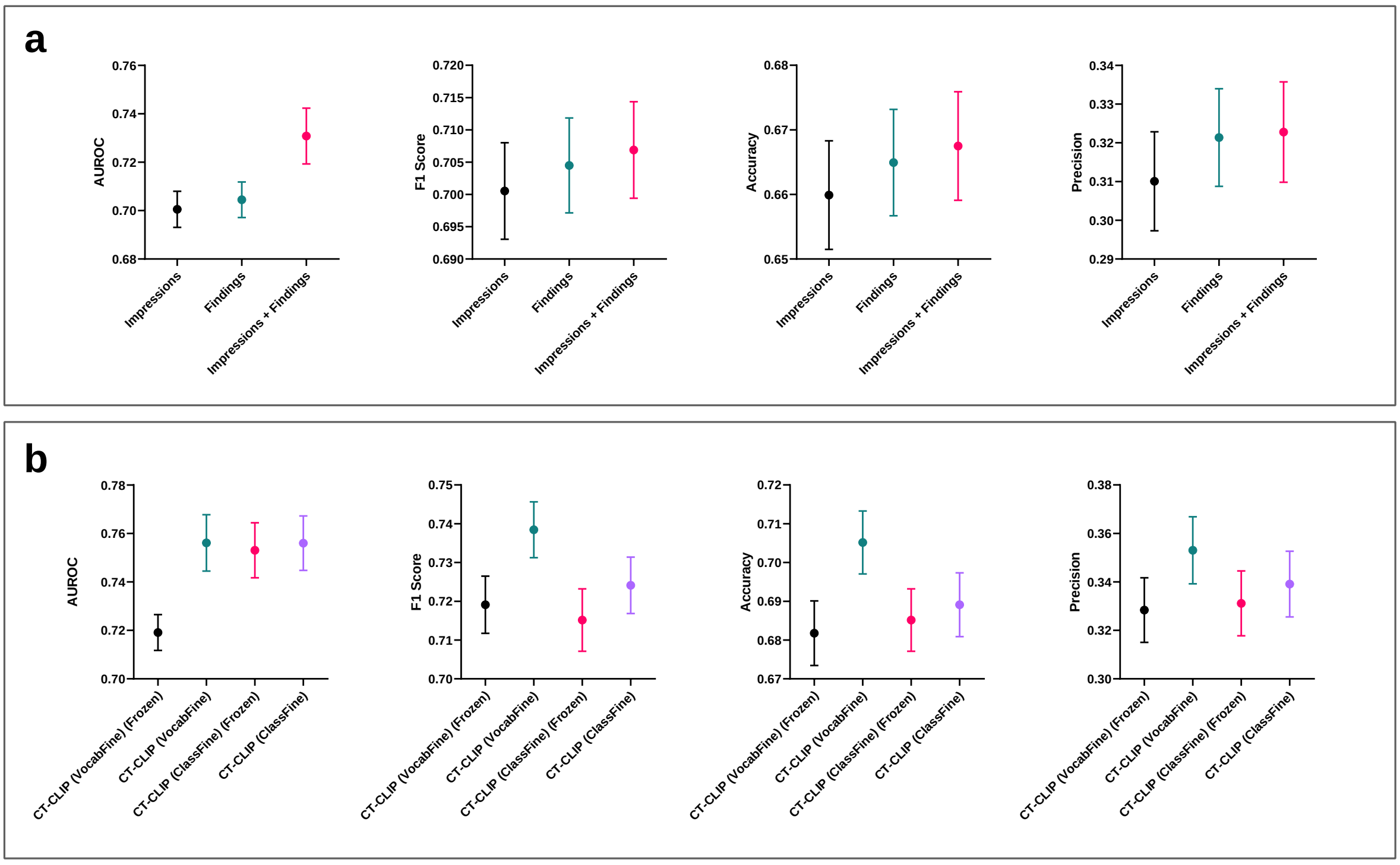

Supplementary Figure 1: Detailed ablation study. a. Compares the zero-shot abnormality detection performance of CT-CLIP models, each using different sections of radiology text reports during training. **b.** Compares various finetuning techniques applied to CT-CLIP models: *frozen* models involve finetuning only the latent layers for the Vocabfine approach, while for the ClassFine method, only the new linear layer is finetuned, leaving all other layers unchanged.

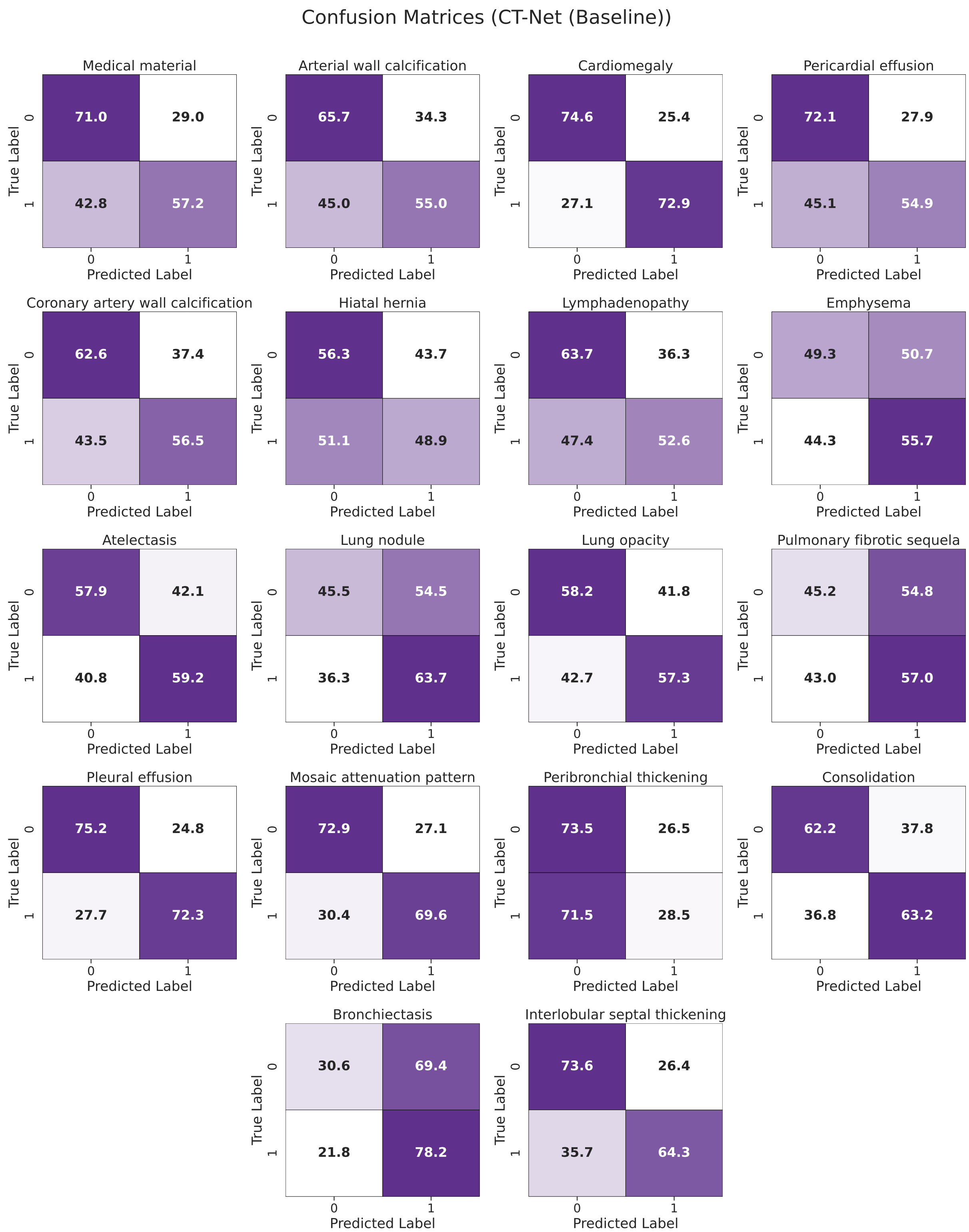

Supplementary Figure 2: Illustration of confusion matrices for the fully supervised baseline model in the internal validation set.

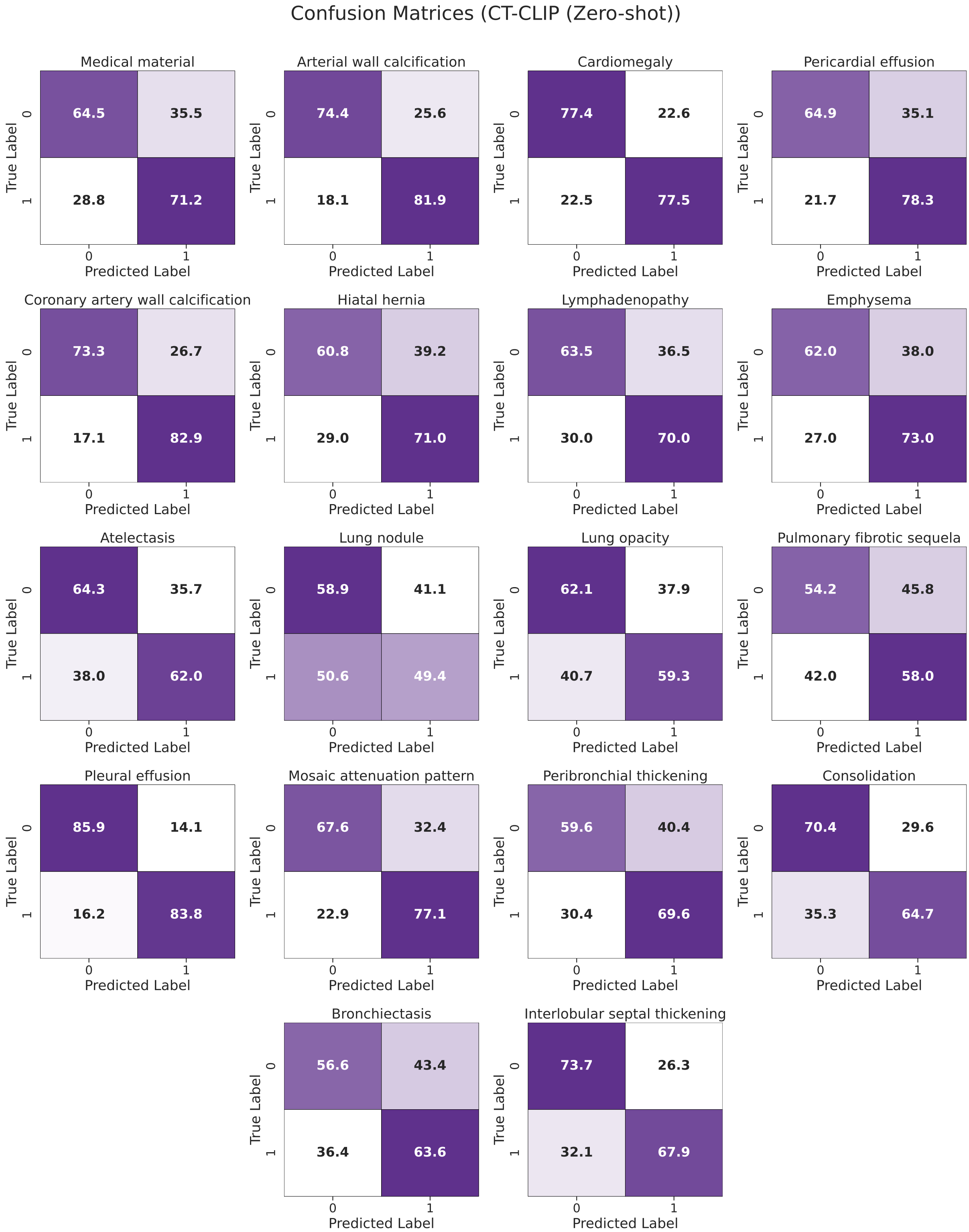

Supplementary Figure 3: Illustration of confusion matrices for the CT-CLIP model in the internal validation set.

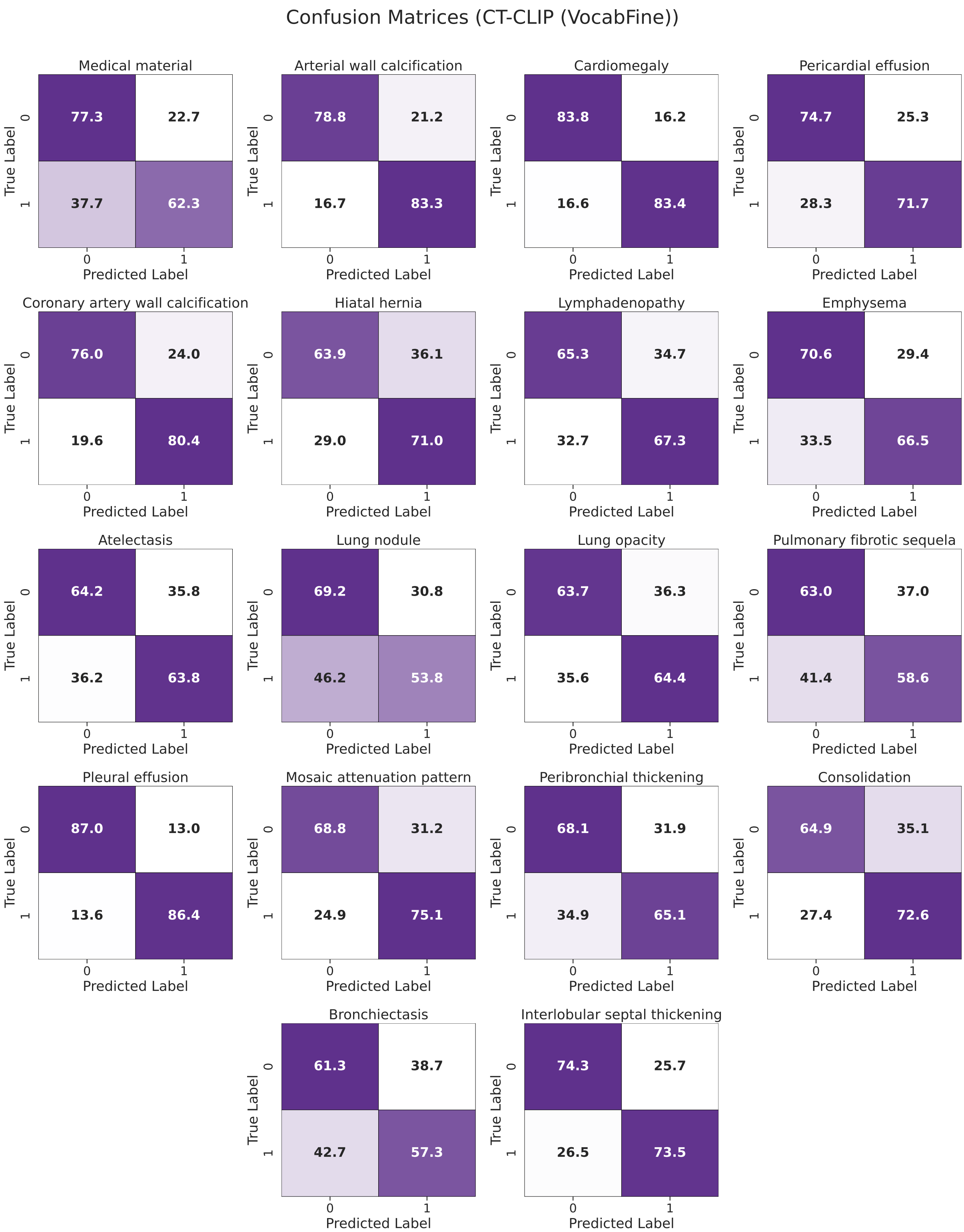

Supplementary Figure 4: Illustration of confusion matrices for the CT-CLIP model fine-tuned with VocabFine in the internal validation set.

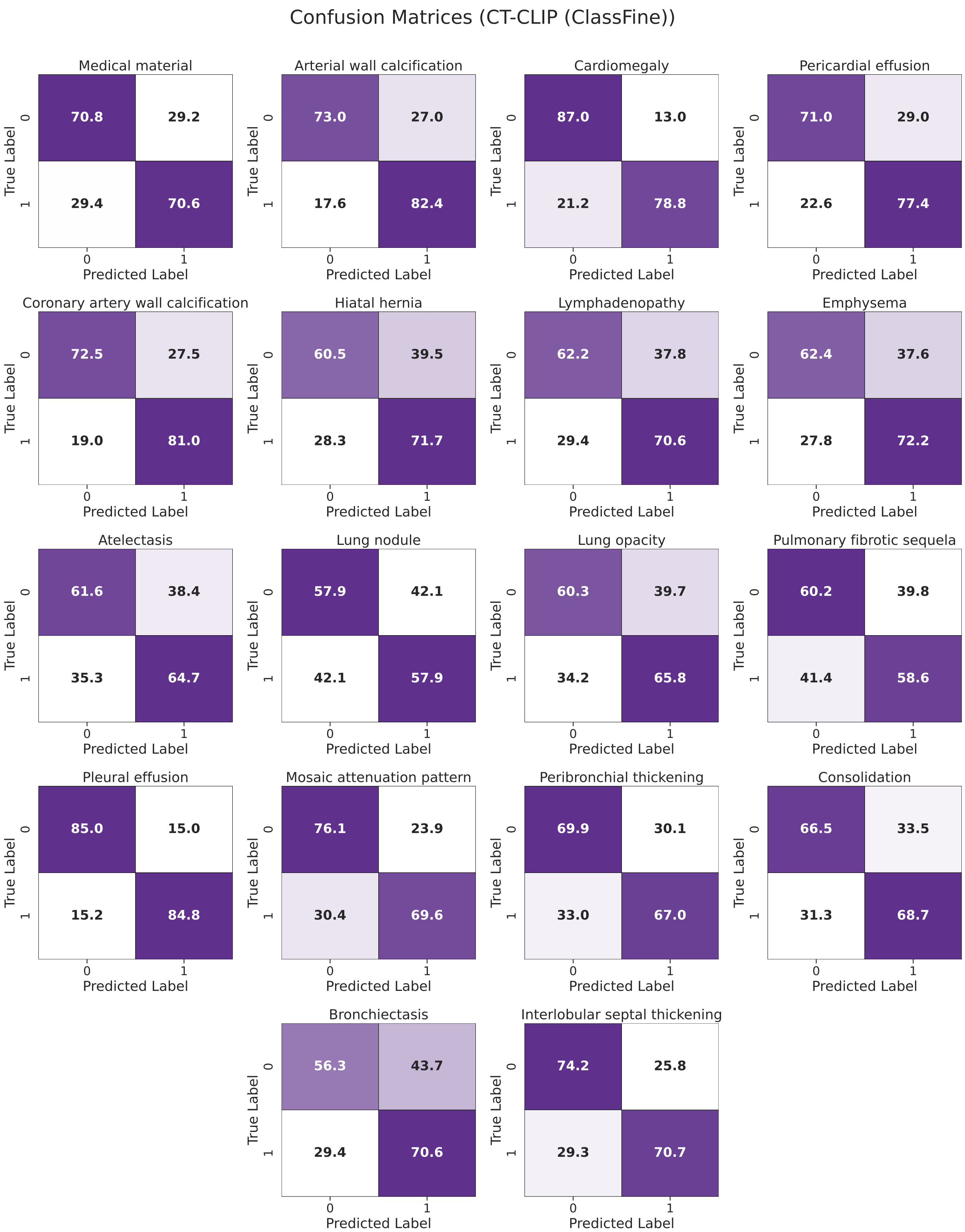

Supplementary Figure 5: Illustration of confusion matrices for the CT-CLIP model fine-tuned with ClassFine in the internal validation set.

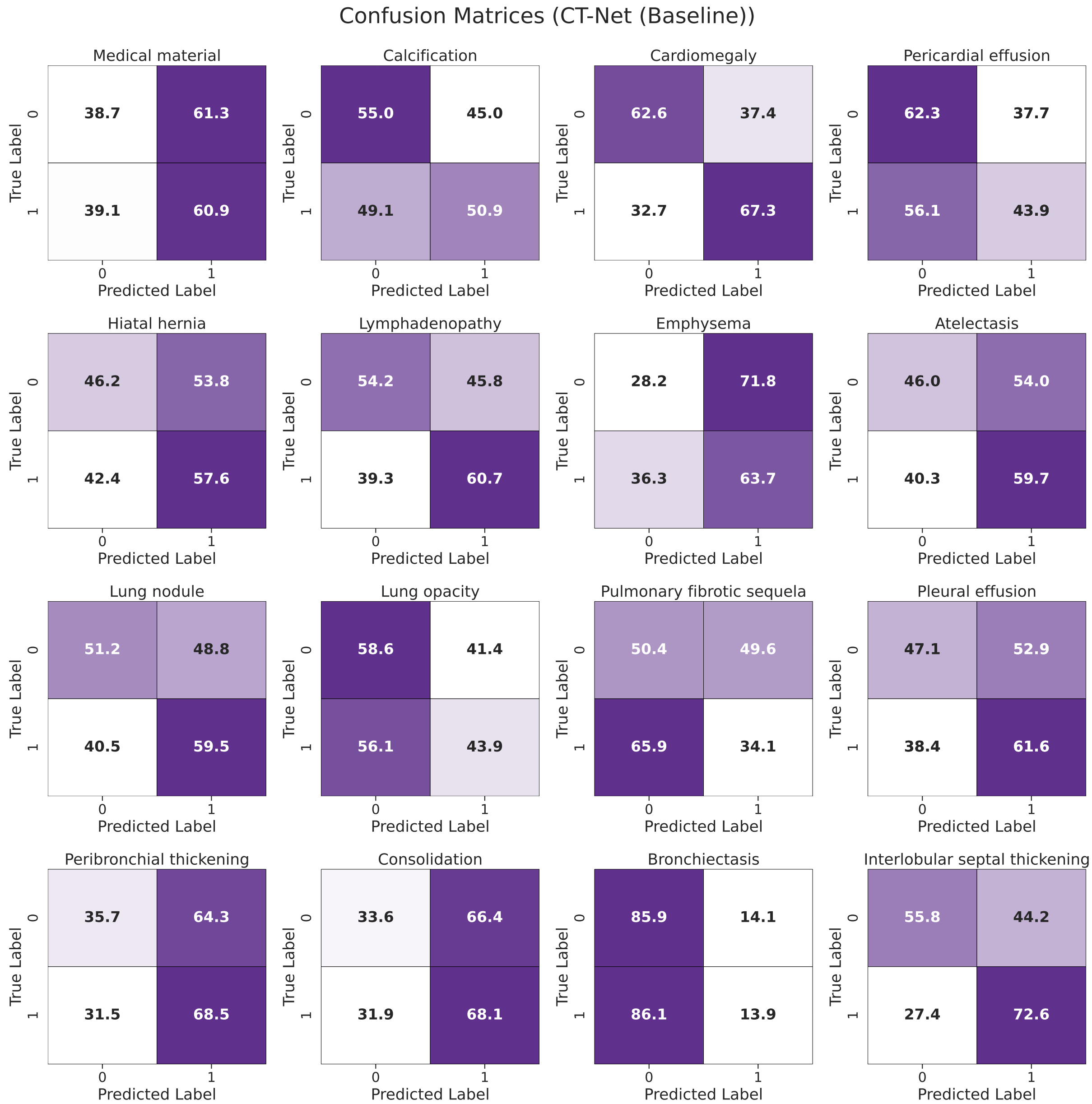

Supplementary Figure 6: Illustration of confusion matrices for the fully supervised baseline model in the RAD-ChestCT external validation set.

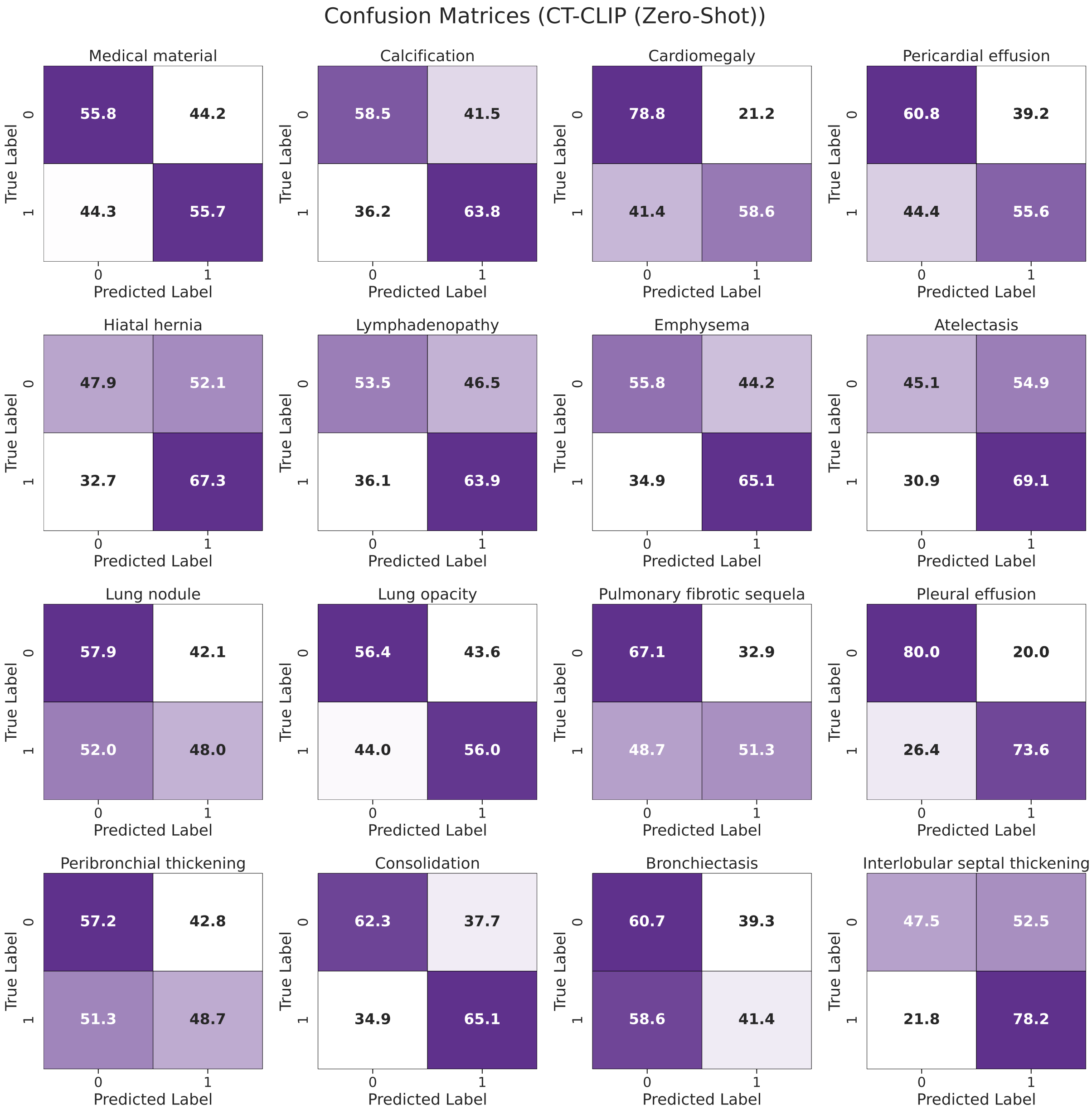

Supplementary Figure 7: Illustration of confusion matrices for the CT-CLIP model in the RAD-ChestCT external validation set.

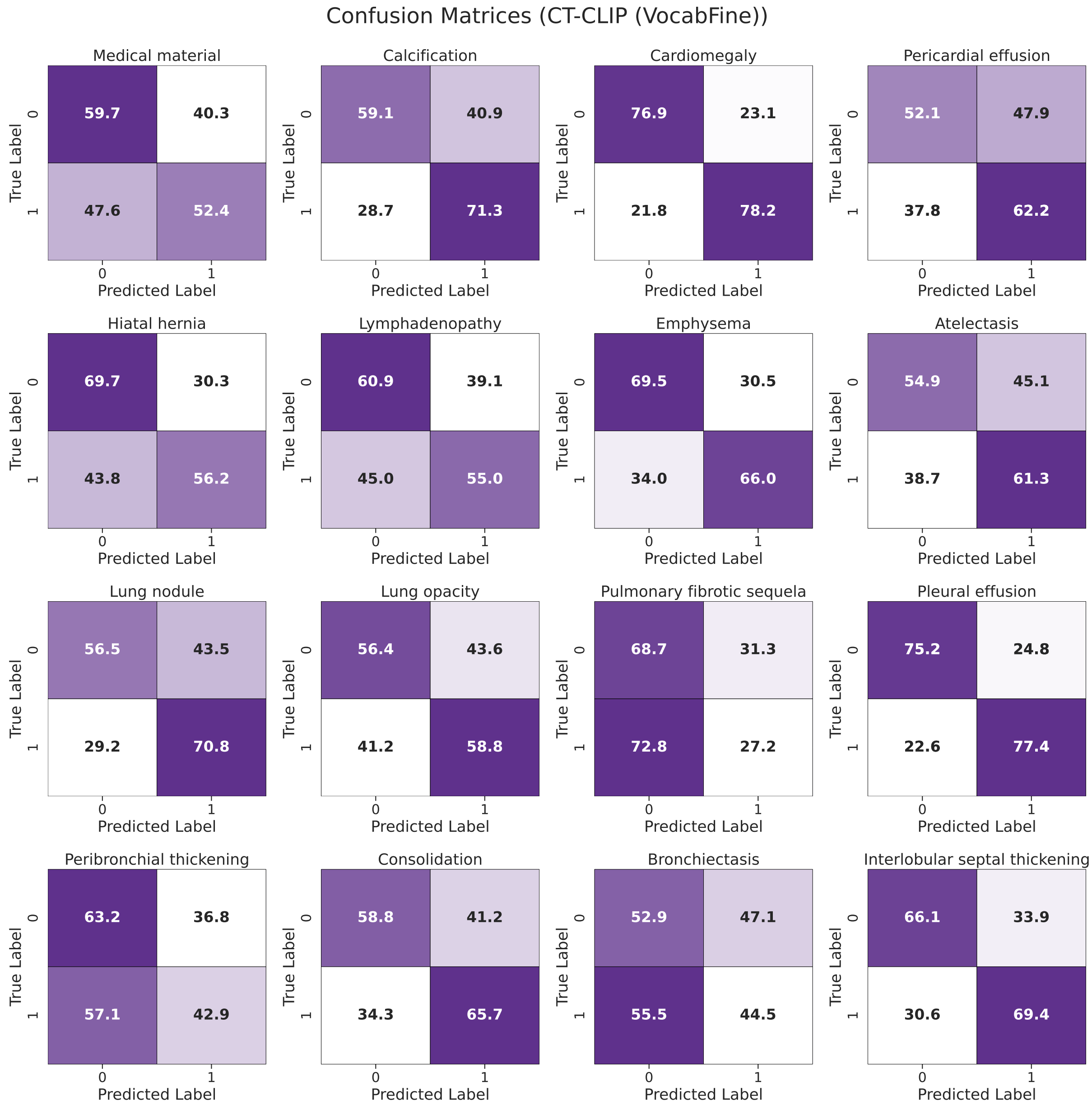

Supplementary Figure 8: Illustration of confusion matrices for the CT-CLIP model fine-tuned with VocabFine in the RAD-ChestCT external validation set.

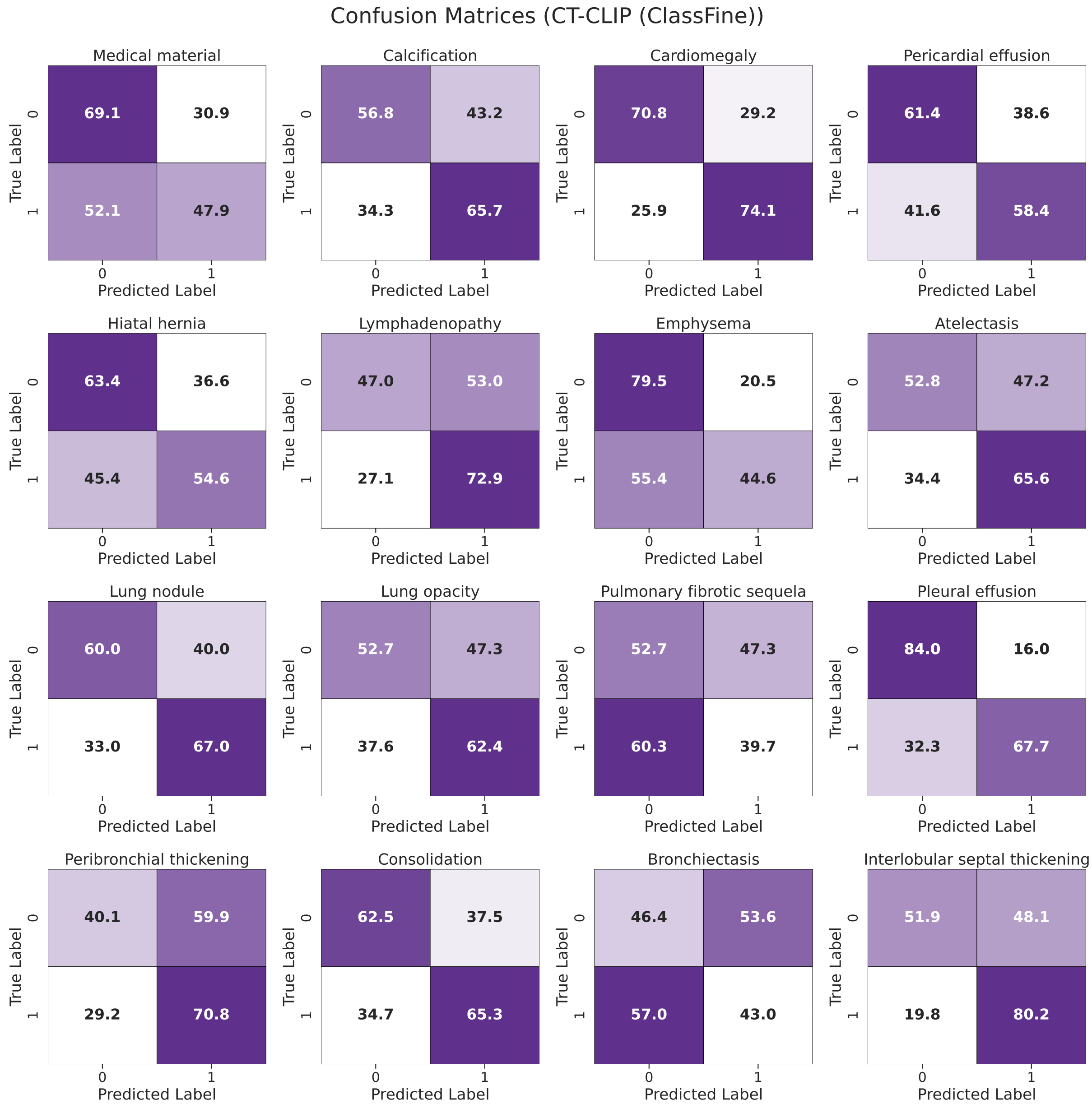

Supplementary Figure 9: Illustration of confusion matrices for the CT-CLIP model fine-tuned with ClassFine in the RAD-ChestCT external validation set.

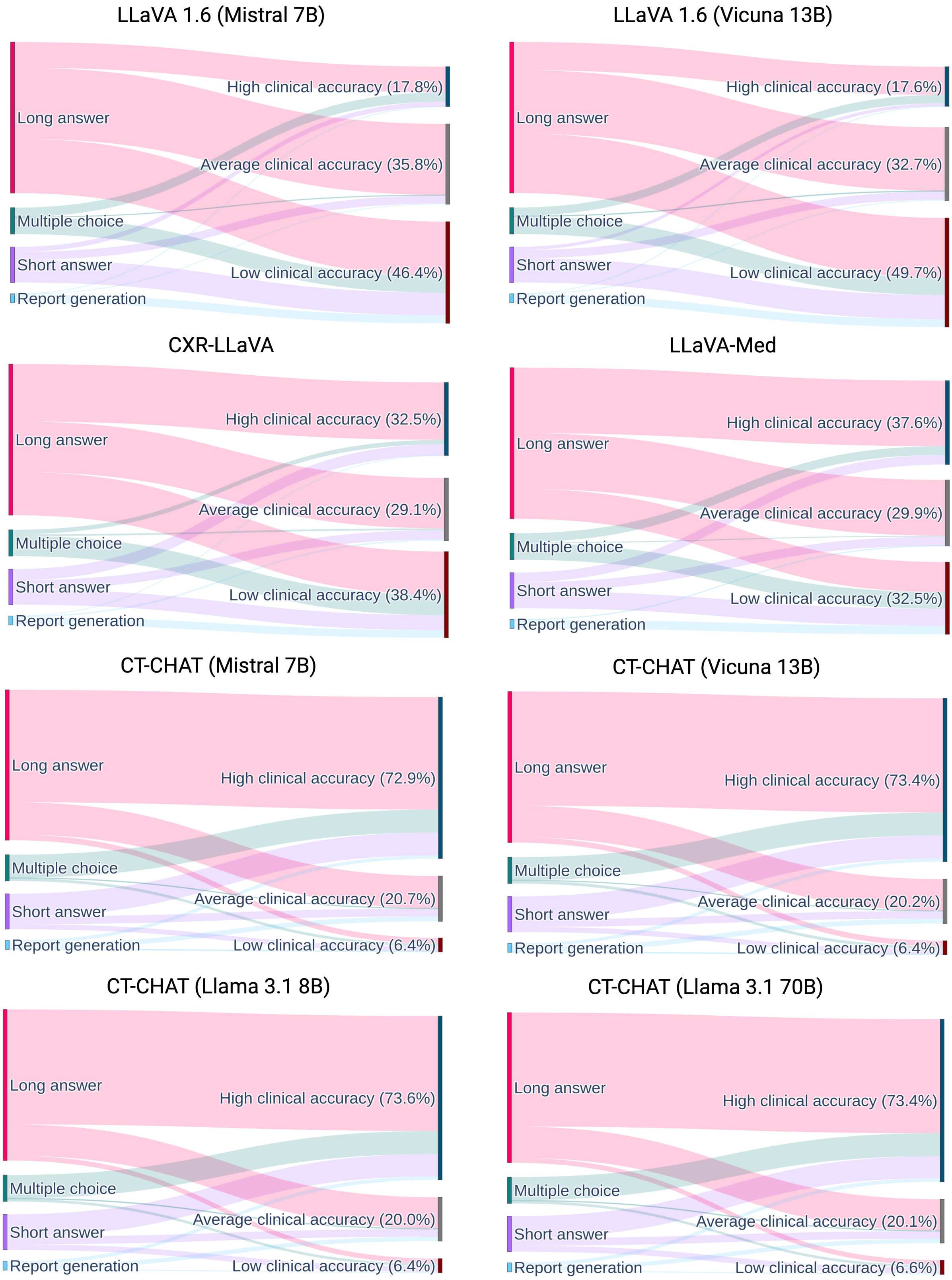

Supplementary Figure 10: Clinical accuracy comparison of VQA models. This figure provides a detailed assessment of clinical accuracy across different models, showing that CT-CHAT produces a higher proportion of outputs in the high clinical accuracy category, indicating superior performance.

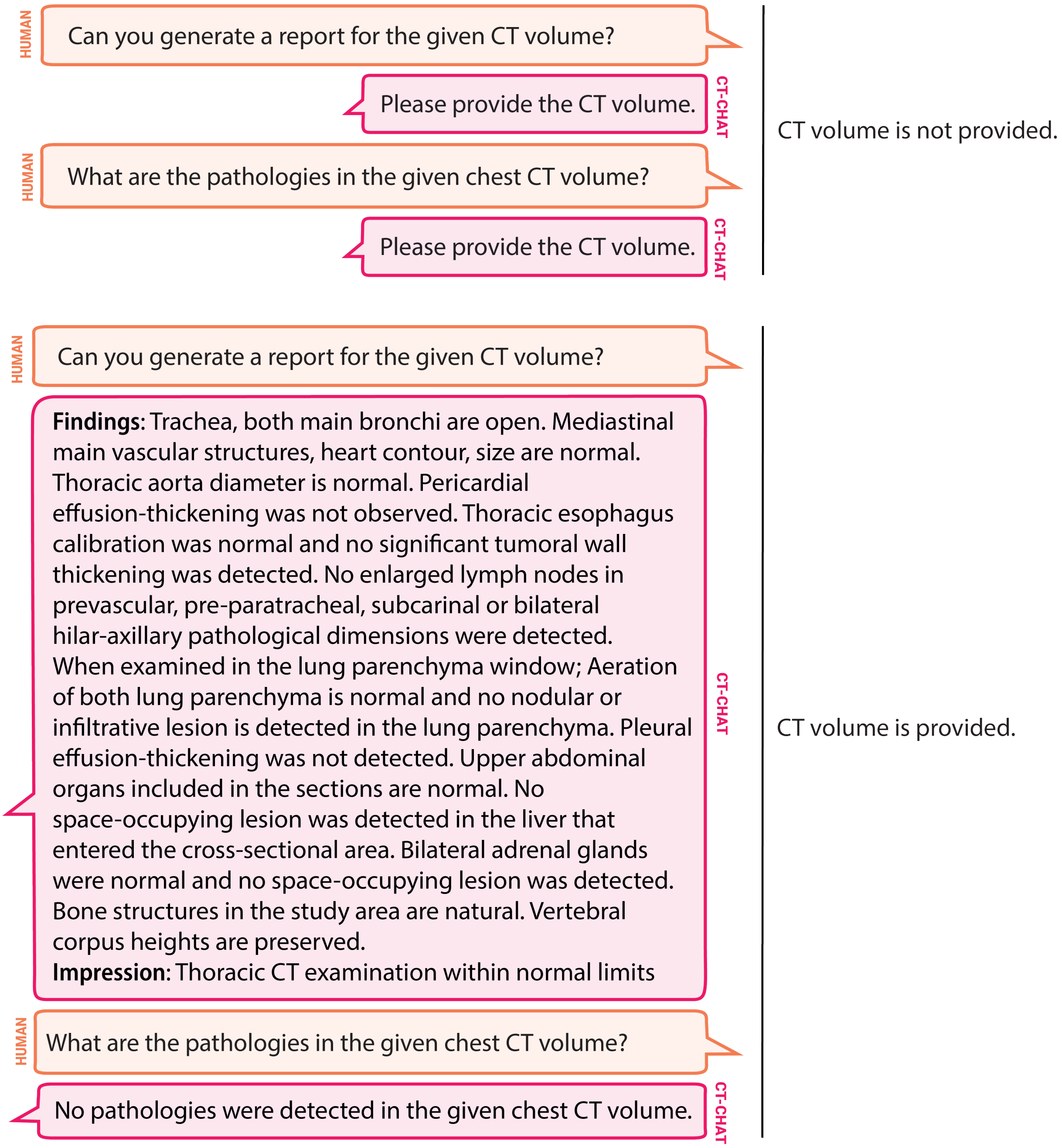

Supplementary Figure 11: Guardrail example to prevent hallucination of CT-CHAT when the CT volume is not provided.
