# Supplementary material for: Generalist foundation models from a multimodal dataset for 3D computed tomography
Source: Nat Biomed Eng. 2026 Feb 12;10(8):1610–28. doi: 10.1038/s41551-025-01599-y (PMC13658014; doi:10.1038/s41551-025-01599-y)
Supplement: Supplementary file 2 [file 41551_2025_1599_MOESM2_ESM.pdf]

nature portfolio

Corresponding author(s): Ibrahim Ethem Hamamci

Last updated by author(s): Sep 20, 2025

Reporting Summary

Nature Portfolio wishes to improve the reproducibility of the work that we publish. This form provides structure for consistency and transparency in reporting. For further information on Nature Portfolio policies, see our Editorial Policies and the Editorial Policy Checklist.

Statistics

For all statistical analyses, confirm that the following items are present in the figure legend, table legend, main text, or Methods section.

n/a	Confirmed	
☐	☒	The exact sample size (*n*) for each experimental group/condition, given as a discrete number and unit of measurement
☐	☒	A statement on whether measurements were taken from distinct samples or whether the same sample was measured repeatedly
☐	☒	The statistical test(s) used AND whether they are one- or two-sided<br>*Only common tests should be described solely by name; describe more complex techniques in the Methods section.*
☐	☒	A description of all covariates tested
☒	☐	A description of any assumptions or corrections, such as tests of normality and adjustment for multiple comparisons
☐	☒	A full description of the statistical parameters including central tendency (e.g. means) or other basic estimates (e.g. regression coefficient) AND variation (e.g. standard deviation) or associated estimates of uncertainty (e.g. confidence intervals)
☐	☒	For null hypothesis testing, the test statistic (e.g. *F*, *t*, *r*) with confidence intervals, effect sizes, degrees of freedom and *P* value noted<br>*Give P values as exact values whenever suitable.*
☒	☐	For Bayesian analysis, information on the choice of priors and Markov chain Monte Carlo settings
☒	☐	For hierarchical and complex designs, identification of the appropriate level for tests and full reporting of outcomes
☒	☐	Estimates of effect sizes (e.g. Cohen's *d*, Pearson's *r*), indicating how they were calculated

Our web collection on statistics for biologists contains articles on many of the points above.

Software and code

Policy information about availability of computer code

Data collection	Scripts to collect data and retrieve images from the PACS server were developed in Python (version 3.9), leveraging the RadiAnt API (RadiAnt DICOM Viewer 2023.1). We specifically retrieved data corresponding to the accession numbers for non-contrast chest CT volumes.
Data analysis	We conducted our experiments using Python and open-source libraries, with full reproducibility ensured through publicly available code. For CT-CLIP, we used Python 3.11.5, along with PyTorch v2.0.1, CUDA v11.7, SciPy v1.11.1, Torchvision v0.15.2, Scikit-learn v1.3.0, Scikit-image v0.20.0, Pandas v2.0.3, NumPy v1.24.4, Transformers v4.30.1, and Accelerate v0.21.1. All preprocessing, training, and analysis scripts for CT-CLIP are available in our GitHub repository, along with environment setup instructions to facilitate replication.<br><br>CT-CLIP training was carried out on a system with four NVIDIA A100 GPUs (80GB each), 500GB of RAM, and 24 CPUs, leveraging Distributed Data Parallelism via the Accelerate library in combination with Fully Sharded Data Parallelism for efficient multi-GPU scaling. Fine-tuning of CT-CLIP, supervised classification experiments, and other downstream tasks were conducted using a single A100 GPU.<br><br>For CT-CHAT, we used a separate environment based on Python 3.12.4, with PyTorch v2.4.0, CUDA v12.4, SciPy v1.14.0, Torchvision v0.19.0, Scikit-learn v1.2.2, Pandas v2.2.2, and NumPy v1.26.4. The largest CT-CHAT model, based on Llama 3.1 70B, was trained using four A100 GPUs with DeepSpeed ZeRO-3 optimization. Other CT-CHAT variants were trained using two A100 GPUs.<br><br>Complete details about our computational environments, required dependencies, and installation instructions are available in our respective GitHub repositories. These resources are designed to ensure transparency and support full reproducibility of our results.

For manuscripts utilizing custom algorithms or software that are central to the research but not yet described in published literature, software must be made available to editors and reviewers. We strongly encourage code deposition in a community repository (e.g. GitHub). See the Nature Portfolio guidelines for submitting code & software for further information.

Data

Policy information about availability of data

All manuscripts must include a data availability statement. This statement should provide the following information, where applicable:

- Accession codes, unique identifiers, or web links for publicly available datasets
- A description of any restrictions on data availability
- For clinical datasets or third party data, please ensure that the statement adheres to our policy

Our CT-RATE dataset, comprising paired non-contrast 3D chest CT volumes, radiology text reports, metadata, multi-abnormality labels, and anatomy-level segmentation masks, is publicly accessible at: https://huggingface.co/datasets/ibrahimhamamci/CT-RATE. Additionally, the first external validation set, RAD-ChestCT, is available at: https://zenodo.org/records/6406114. The trained models and source code are available in our GitHub repository: https://github.com/ibrahimethemhamamci/CT-CLIP and https://github.com/ibrahimethemhamamci/CT-CHAT. Unfortunately, the second external validation set, UPMC, is not publicly available, as it is a local and private test set used by our external collaborators for independent evaluation.

Research involving human participants, their data, or biological material

Policy information about studies with human participants or human data. See also policy information about sex, gender (identity/presentation), and sexual orientation and race, ethnicity and racism.

Reporting on sex and gender	In our CT-RATE dataset, derived from retrospective medical records, the patient sex distribution includes 41.6% female and 58.4% male among 21,304 unique individuals. This dataset does not utilize patient sex as an analytical covariate. The first external validation set, RAD-ChestCT, does not include sex information. Unfortunately, for the second external validation set, UPMC, being a private dataset, we do not have access to metadata and are therefore unable to disclose any such details. Our analysis and model development focus primarily on technological advancements in medical image interpretation, without direct consideration of sex or gender impacts.
Reporting on race, ethnicity, or other socially relevant groupings	Our study does not include race, ethnicity, or other socially relevant groupings as part of the dataset analysis. The CT-RATE dataset, derived retrospectively from existing medical records at Istanbul Medipol University Mega Hospital, does not contain information on race or ethnicity, and such variables were not collected or considered during the design, preprocessing, or analysis phases. Similarly, the external validation datasets—RAD-ChestCT (Duke University Hospital, USA) and UPMC (University of Pittsburgh Medical Center, USA)—do not provide access to race or ethnicity metadata. As such, no subgroup analyses were performed based on these variables, and they were not used as covariates in model development or evaluation. Our focus remained on technical and diagnostic performance, independent of social groupings.
Population characteristics	The CT-RATE dataset includes 21,304 unique adult patients ranging in age from 18 to 102 years, with a mean age of 48.8 years and the most common age being 40 years. The dataset comprises 41.6% female and 58.4% male patients. These population characteristics are included as part of the demographic profile for each case, but were not used as analytical covariates in our study. Diagnoses were extracted from free-text radiology reports using automated labeling tools, with detailed statistics provided in the manuscript. The first external validation set, RAD-ChestCT, originates from Duke University Hospital in the United States and represents a demographically and geographically distinct patient cohort. While sex and age metadata are unavailable in RAD-ChestCT, multi-abnormality diagnostic labels are provided and used for evaluation, as described in the manuscript. The second external validation set, UPMC, was obtained from the University of Pittsburgh Medical Center (USA) and constitutes a private dataset. Due to data use restrictions, we do not have access to any demographic or diagnostic metadata from this cohort. Nevertheless, this dataset was used for fully independent validation of our models in a distinct clinical setting, highlighting the generalizability.
Recruitment	Patient recruitment was unnecessary for the retrospective use of chest CT volumes and associated radiology text reports.
Ethics oversight	Our study received ethical approval from the Clinical Research Ethics Committee at Istanbul Medipol University (E-10840098-772.02-6841, 27/10/2023) for the open-sourcing of our CT-RATE dataset and associated trained models.

Note that full information on the approval of the study protocol must also be provided in the manuscript.

Field-specific reporting

Please select the one below that is the best fit for your research. If you are not sure, read the appropriate sections before making your selection.

☒ Life sciences ☐ Behavioural & social sciences ☐ Ecological, evolutionary & environmental sciences

For a reference copy of the document with all sections, see nature.com/documents/nr-reporting-summary-flat.pdf

Life sciences study design

All studies must disclose on these points even when the disclosure is negative.

Sample size	No specific sample size calculations were performed prior to the creation of CT-RATE. Instead, our dataset curation strategy aimed to include the most c.mprehensive collection of non-contrast chest CT volumes and corresponding radiology reports available from Istanbul Medipol University Mega Hospital. Spanning data acquired between May 2015 and January 2023, we collected a total of 50,188 reconstructed 3D chest CT volumes, derived from 25,692 distinct CT scans conducted on 21,304 unique patients. The final sample size was determined by the

	full extent of relevant data accessible in the hospital's database at the time of dataset creation. Further details on this dataset, including information on the external validation sets, can be found in the "Creating the CT-RATE dataset" subsection of the Methods section.
Data exclusions	No data were excluded from the CT-RATE dataset following its initial curation.
Replication	We confirm that all our experiments can be reproduced using the provided source code and datasets.
Randomization	In our CT-RATE dataset, 20,000 patients were randomly assigned to the training set and 1,304 patients to the validation set, ensuring that chest CT volumes from the same patient appear exclusively in only one of the sets to preserve data integrity. For the first external validation set, we utilized all publicly available data from RAD-ChestCT, which does not include any patient-identifiable or demographic information. For the second external validation set, UPMC, a private dataset from a different country and patient cohort, we do not have access to metadata and therefore cannot disclose any demographic or diagnostic details.
Blinding	Blinding was unnecessary for our study, as it relied on anonymized non-contrast chest CT volumes and associated radiology text reports.

Reporting for specific materials, systems and methods

We require information from authors about some types of materials, experimental systems and methods used in many studies. Here, indicate whether each material, system or method listed is relevant to your study. If you are not sure if a list item applies to your research, read the appropriate section before selecting a response.

Materials & experimental systems

n/a	Involved in the study
☒	☐ Antibodies
☒	☐ Eukaryotic cell lines
☒	☐ Palaeontology and archaeology
☒	☐ Animals and other organisms
☒	☐ Clinical data
☒	☐ Dual use research of concern
☒	☐ Plants

Methods

n/a	Involved in the study
☒	☐ ChIP-seq
☒	☐ Flow cytometry
☒	☐ MRI-based neuroimaging

Plants

Seed stocks	*Report on the source of all seed stocks or other plant material used. If applicable, state the seed stock centre and catalogue number. If plant specimens were collected from the field, describe the collection location, date and sampling procedures.*
Novel plant genotypes	*Describe the methods by which all novel plant genotypes were produced. This includes those generated by transgenic approaches, gene editing, chemical/radiation-based mutagenesis and hybridization. For transgenic lines, describe the transformation method, the number of independent lines analyzed and the generation upon which experiments were performed. For gene-edited lines, describe the editor used, the endogenous sequence targeted for editing, the targeting guide RNA sequence (if applicable) and how the editor was applied.*
Authentication	*Describe any authentication procedures for each seed stock used or novel genotype generated. Describe any experiments used to assess the effect of a mutation and, where applicable, how potential secondary effects (e.g. second site T-DNA insertions, mosiacism, off-target gene editing) were examined.*
