# Supplementary material for: Generalist foundation models from a multimodal dataset for 3D computed tomography
Source: Nat Biomed Eng. 2026 Feb 12;10(8):1610–28. doi: 10.1038/s41551-025-01599-y (PMC13658014; doi:10.1038/s41551-025-01599-y)
Supplement: Supplementary file 3 [file 41551_2025_1599_MOESM3_ESM.pdf]

nature portfolio

Peer Review File

Generalist Foundation Models from a Multimodal Dataset for 3D Computed Tomography

Corresponding Author: Dr Ibrahim Ethem Hamamci

This manuscript has been previously reviewed at another journal. This document only contains information relating to versions considered at Nature Biomedical Engineering.

Version 0:

Decision Letter:

Dear Ibrahim,

Thank you again for submitting to *Nature Biomedical Engineering* your manuscript, "Developing Generalist Foundation Models from a Multimodal Dataset for 3D Computed Tomography", and for your patience in waiting for the reviewer reports and our feedback. The manuscript has been seen by two experts, whose reports you will find at the end of this message. You will see that the reviewers appreciate the work, and that they raise a number of technical criticisms that we hope you will be able to address. In particular, we would expect that a revised version of the manuscript provides:

* Extended performance comparisons with more recent generative models for tasks in computed tomography, and including ablation-based analyses, as suggested by Reviewer #1.

* Evidence of the utility of the model in additional tasks, and with CT scans of other body parts and pathologies.

* Further external validation, as argued by Reviewer #1.

When you are ready to resubmit your manuscript, please upload the revised files, a point-by-point rebuttal to the comments from all reviewers, the reporting summary, and a cover letter that explains the main improvements included in the revision and responds to any points highlighted in this decision.

Please follow the following recommendations:

* Clearly highlight any amendments to the text and figures to help the reviewers and editors find and understand the changes (yet keep in mind that excessive marking can hinder readability).

* If you and your co-authors disagree with a criticism, provide the arguments to the reviewer (optionally, indicate the relevant points in the cover letter).

* If a criticism or suggestion is not addressed, please indicate so in the rebuttal to the reviewer comments and explain the reason(s).

* Consider including responses to any criticisms raised by more than one reviewer at the beginning of the rebuttal, in a section addressed to all reviewers.

* The rebuttal should include the reviewer comments in point-by-point format (please note that we provide all reviewers will the reports as they appear at the end of this message).

* Provide the rebuttal to the reviewer comments and the cover letter as separate files.

We expect that you will be able to resubmit the manuscript within 12 weeks of receiving this message. If this is the case, you will be protected against potential scooping. Otherwise, we will be happy to consider a revised manuscript as long as the significance of the work is not compromised by work published elsewhere or accepted for publication at *Nature Biomedical*

Engineering.

We hope that you will find the referee reports helpful when revising the work, which we look forward to receive. Please do not hesitate to contact me should you have any questions.

Best wishes,

Pep

__
Pep Pàmies
Chief Editor, *Nature Biomedical Engineering*

____
Reviewer #1 (Report for the authors (Required)):

The authors have presented a promising foundational model for 3D chest CT and released an open-source dataset that includes CT volumes and associated reports. The structured code repository is highly commendable and provides transparency for replication and further development. The methods and results appear scientifically sound, and the model shows potential for advancing research in the domain. However, there are several areas where the manuscript could be improved, especially in the background analysis, model evaluation, and result reporting.

The authors only compare their CT-Clip model with CT-Net, a model from 2020. While this comparison is valuable, it is somewhat outdated. Given the rapid advancements in medical image analysis, I strongly encourage the authors to extend their analysis to more recent models. One such example is MedSyn, which has demonstrated impressive performance in similar tasks. A more comprehensive comparison with recent models would provide a clearer context for evaluating the effectiveness of the proposed model.

The manuscript refers to a "CT foundational model," but it is trained solely on chest CT scans and validated on chest abnormalities. This raises a question about the broader applicability of the model. Given the ambitious terminology of "foundational," it would be helpful if the authors could either refine the objective to focus exclusively on chest CT or expand the model's validation to include other anatomical regions. If the latter approach is taken, this should be clearly stated in the manuscript to avoid ambiguity regarding the model’s generalizability.

The validation is performed on a single external dataset. While this is useful, it limits the robustness of the conclusions drawn. I recommend that the authors expand their validation efforts by including the M3D dataset, which is more diverse and includes a broader range of cases. Additionally, it would be valuable to report the model’s zero-shot performance on both common and rare diseases separately, which would offer a more nuanced understanding of the model’s capabilities and limitations.

The manuscript would benefit from a thorough ablation study to highlight the contributions of various design choices. For example, the impact of including different sections of the reports, the effect of volume resizing, and the comparative performance of vocabfine vs classfine should all be systematically analyzed. Such an ablation study would provide a deeper understanding of the model’s strengths and limitations, as well as guidance for future improvements.

Reviewer #2 (Report for the authors (Required)):

Summary
The manuscript presents CT-RATE, a large-scale open-source dataset pairing 3D chest CT volumes with radiology reports. This dataset serves as the foundation for CT-CLIP, a model leveraging contrastive learning for abnormality detection and case retrieval in 3D medical CT imaging. Additionally, the authors develop CT-CHAT, a vision-language AI assistant fine-tuned on CT-CLIP embeddings. The study demonstrates superior zero-shot performance compared to supervised baselines, establishing a new benchmark in the field of 3D CT medical imaging.

Strength
-The dataset represents a major advancement in CT-related AI research, akin to the role of paired image-report datasets in 2D imaging. The scale and complexity of the dataset, including the effort to process, deidentification, and secure ethical approval, make this quite a valuable resource. CT-CLIP can serve as a valuable baseline for further studies that integrates CT and reports.
-CT-CLIP offers a solid baseline model for integrating CT imaging with textual data, potentially driving future advancements in the field. Descent amount of experiments backups the concept of this model.
-The demonstrated adaptability of CT-CLIP to MLLMs highlights its potential for future expansions and applications in multimodal AI.
- The open-source availability of the dataset and code ensures reproducibility and supports further research.

Weakness
-(Minor Suggestion) : The manuscript could detail the trade-offs between ClassFine and VocabFine approaches to better inform their application in specific use cases. Such as the reason why these finetuning methods are used to evaluate retrieval tasks are not clear.

-(Minor Suggestion) : The inclusion of experiments related to detection and segmentation tasks, which are critical in CT imaging, would enhance the study's practical relevance.

-(Minor Suggestion) : As CLIP and VLM models aim to extract rich representations independent of predefined supervised classes, a demonstration of fine-tuning with a smaller portion of the data could provide valuable insights into the model's efficiency and adaptability.

-For Figure 6, metrics such as BLEU and ROUGE are text-focused and may inherently favor the model due to differences in training modalities. Adding a clinical relevance metric—similar to an adaptation of ChexBert F1 for CXR—would provide a more reliable assessment of report informativeness. The authors can use their own trained report labeler model for this.

-While the approach itself is not highly novel, the paper's focus on creating a baseline model and dataset which is acceptable given its intended purpose to establish a foundation for further advancements.

The authors have conducted a well-rounded study, creating the first vision-language model and dataset tailored for VLM in 3D chest CT imaging. While the technical novelty is limited, the dataset and baseline model introduced here are ready to have a transformative effect on the field.

Version 1:

Decision Letter:

Dear Dr Hamamci,

Your manuscript "Developing Generalist Foundation Models from a Multimodal Dataset for 3D Computed Tomography" has now been seen by 2 reviewers. In light of their advice and on the basis of our editorial evaluation, I would be happy to consider a revised version of the manuscript.

The reviewers have some minor comments that we would ask that you address before we move ahead with acceptance of the paper.

As before, when you are ready to resubmit your manuscript, please upload the revised files, a point-by-point rebuttal to the comments from all reviewers, and a cover letter that explains the main improvements included in the revision and responds to any points highlighted in this decision.

As a reminder, please follow the following recommendations:

* Please submit your revised manuscript in a word doc or LaTeX format.

* Clearly highlight any amendments to the text and figures to help the reviewers and editors find and understand the changes (yet keep in mind that excessive marking can hinder readability).

* If you and your co-authors disagree with a criticism, provide the arguments to the reviewer (optionally, indicate the relevant points in the cover letter).

* If a criticism or suggestion is not addressed, please indicate so in the rebuttal to the reviewer comments and explain the reason(s).

* Consider including responses to any criticisms raised by more than one reviewer at the beginning of the rebuttal, in a section addressed to all reviewers.

* The rebuttal should include the reviewer comments in point-by-point format (please note that we provide all reviewers will the reports as they appear at the end of this message).

* Provide the rebuttal to the reviewer comments and the cover letter as separate files.

We expect that you will be able to resubmit the manuscript within 8 weeks of receiving message. If this is the case, you will be protected against potential scooping. Otherwise, we will be happy to consider the next version of the manuscript as long as the advance of the work is not compromised by work published elsewhere or accepted for publication at *Nature Biomedical Engineering*.

We look forward to receive a further revised version of the work. Please do not hesitate to contact me should you have any questions.

Best wishes,

Barbara Cheifet
Editor
Nature Biomedical Engineering

____
Reviewer #1 (Report for the authors (Required)):

The authors have addressed my concerns and conducted an additional experiment using an external dataset. They have also expanded the comparative performance analysis.

I sincerely appreciate the authors' effort in making the data and code publicly available.

However, I believe it is essential to have a minimum of three clinical experts review the quality of the reports and chat outputs, specifically assessing comprehensiveness, hallucinations, and missing information, before the manuscript is considered for publication.

Reviewer #2 (Report for the authors (Required)):

Summary:
The authors present CT-RATE, an open-source dataset comprising over 25,000 3D chest CT scans paired with radiology reports annotated for 18 abnormalities. Building on this, they introduce CT-CLIP, a vision-language model for 3D chest CTs, and CT-CHAT, a multimodal assistant capable of answering questions and generating reports from chest CT data. The paper demonstrates that CT-CLIP achieves strong performance in multi-abnormality detection and retrieval, and that CT-CHAT is a promising step toward advanced AI-driven report generation.

--Degree of Advance
CT-RATE Dataset: The release of CT-RATE fills a significant gap, providing the largest publicly available, open-source dataset of paired 3D chest CT scans and radiology reports. This resource will be invaluable for the research community and enables a new scale of multimodal medical AI research.

CT-CLIP Model: CT-CLIP, trained on CT-RATE, have the potential to improve representation learning for chest CT and deliver robust performance on various downstream tasks, establishing a new methodological standard in the field.

--Broad Implications.
- CT-RATE is likely to serve as a launchpad for rapid development of chest CT-related AI models and could spark extensive benchmarking and follow-up studies in the field.

Major comments:
1. The absence of a wider variety of downstream tasks (such as segmentation and detection) limits the demonstration of the model’s representational power. However, this is understandable given the current lack of public data with such annotations. Could the authors consider adapting a downstream task using LUNA16 (for nodule detection), to further demonstrate model versatility?

Minor comments:
1. For transparency and scientific integrity, I recommend that the authors clarify whether the manuscript was originally drafted in English or translated from another language. If translation was involved at any stage, please provide details on the process and whether professional translation or native English editing was used.

2. While it’s good to see clinical F1, precision, and recall metrics included for report generation, the actual F1 scores are still quite low. This shows that, even though there’s been improvement compared to previous models, the report generation is still far from being ready for clinical use. I suggest that the authors talk more about this limitation in the paper and share some ideas or plans for how this could be improved in the future.

Optional Suggestions.
1. The open-access dataset’s image files are very large. Providing a version with reduced image size or compressed data

types would be extremely helpful to many researchers and could increase accessibility.

Reviewer #2 (Remarks on code availability):

Although there are some minor issues in the code, they do not prevent its use, and it should be straightforward for others to retrain the models if needed.

Version 2:

Decision Letter:

Dear Dr Hamamci,

Thank you for your revised manuscript, "Developing Generalist Foundation Models from a Multimodal Dataset for 3D Computed Tomography". I am pleased to write that we shall be happy to publish the manuscript in *Nature Biomedical Engineering*.

We will be performing detailed checks on your manuscript, and in due course will send you a checklist detailing our editorial and formatting requirements. You will need to follow these instructions before you upload the final manuscript files.

Please do not hesitate to contact me if you have any questions.

Best wishes,

Barbara Cheifet
Editor
Nature Biomedical Engineering

____

Version 3:

Decision Letter:

Dear Dr Hamamci,

I am happy to inform you that your manuscript, "Generalist Foundation Models from a Multimodal Dataset for 3D Computed Tomography", has now been accepted for publication in *Nature Biomedical Engineering*.

Over the next few weeks, the figures will be checked for production quality, the text edited to ensure that it conforms to house style, and the manuscript typeset.

Our Articles are published about 40 days after the acceptance date (we recommend that you inform your institutional press office of this timeframe), and you will be notified of the actual publication date a few days in advance. Articles can be published any working day of the week, and are pushed live shortly after 10 am London time.

Publishing agreement. You will be asked to digitally sign a publishing agreement (grant of rights). After the signed publishing agreement has been received, the proofs of the article will be sent to you for review. If you have any queries during the production process, or you cannot meet the requested deadline for returning the proofs, please contact rjsproduction@springernature.com.

Nature Biomedical Engineering is a Transformative Journal. Authors may publish their research with us through the traditional subscription access route, or make their paper immediately open access through payment of an article-processing charge. More information about publication options is available.

You may need to take specific actions to comply with funder and institutional open-access mandates. If the work described in the accepted manuscript is supported by a funder that requires immediate open access (as outlined, for

example, by Plan S) and your manuscript was originally submitted on or after January 1st 2021, then you should select the gold OA route. Authors selecting subscription publication will need to accept our standard licensing terms (including our self-archiving policies), and these will supersede any other terms that the author or any third party may assert apply to any version of the manuscript.

Acceptance of your manuscript is conditional on agreement, by all authors, with both our media embargo and confidentiality and pre-publicity policies. In particular, you may arrange your own publicity of the Article (for instance, through your institutional press office), as long as you ensure that journalists strictly adhere to the media embargo.

To assist you in disseminating the work, as soon as the Article is published you will be able to take advantage of the Springer Nature SharedIt initiative to generate a unique shareable link to the Article that will allow anyone (with or without a subscription) to read it. Recipients of the link who are subscribers will also be able to download and print the PDF.

Thank you for having submitted this work to *Nature Biomedical Engineering*.

Best wishes,

Barbara Cheifet
Editor
Nature Biomedical Engineering

Open Access This Peer Review File is licensed under a Creative Commons Attribution 4.0 International License, which permits use, sharing, adaptation, distribution and reproduction in any medium or format, as long as you give appropriate credit to the original author(s) and the source, provide a link to the Creative Commons license, and indicate if changes were made.
In cases where reviewers are anonymous, credit should be given to 'Anonymous Referee' and the source.
The images or other third party material in this Peer Review File are included in the article’s Creative Commons license, unless indicated otherwise in a credit line to the material. If material is not included in the article’s Creative Commons license and your intended use is not permitted by statutory regulation or exceeds the permitted use, you will need to obtain permission directly from the copyright holder.
To view a copy of this license, visit https://creativecommons.org/licenses/by/4.0/

Rebuttal to Reviewer #1

Comment 1: Comparison limited to CT-Net, inclusion of MedSyn

Reviewer: The authors only compare their CT-CLIP model with CT-Net, a model from 2020. While this comparison is valuable, it is somewhat outdated. Given the rapid advancements in medical image analysis, I strongly encourage the authors to extend their analysis to more recent models. One such example is MedSyn, which has demonstrated impressive performance in similar tasks. A more comprehensive comparison with recent models would provide a clearer context for evaluating the effectiveness of the proposed model.

Response:
We appreciate the reviewer's observation and fully agree that a broader comparison can better contextualize the performance of our proposed model. While CT-Net remains one of the very few publicly benchmarked methods for multi-abnormality detection in 3D chest CT, the field has indeed advanced significantly since its release. However, due to the historical lack of publicly available 3D chest CT datasets paired with radiology reports, very few follow-up models in this specific area have been published—highlighting the critical need and novelty of our CT-RATE dataset. We thank the reviewer for mentioning MedSyn. Although MedSyn addresses a different task—namely text-conditional 3D CT generation—it shares a similar vision-language paradigm and is built on a comparable dataset. While their dataset is private, we reached out to the authors and were fortunate to collaborate with them for external validation. They kindly evaluated our CT-CLIP model on their in-house test set, and we now report the results in **Figure 4a**, with additional context in the *"Surpassing supervised levels with CT-CLIP's zero-shot multi-abnormality detection"* section of the Results and *"Adding external validation datasets to CT-RATE"* in the Methods.

In response to this suggestion, we also decided to compare **CT-CHAT**, our vision-language assistant, with **more recent 3D report generation models**, including **RadFM** and **CT2Rep** (also raised by Reviewer #2), using clinically meaningful diagnostic metrics (e.g., precision, recall, and F1). CT-CHAT, although primarily designed for VQA, surprisingly outperforms these specialized report generation models (see **Figure 6e** and *"Surpassing other multimodal AI assistants with CT-CHAT"* in the Results). Notably, CT2Rep was trained on the same CT-RATE dataset, but lacks the benefit of contrastive pretraining, which highlights the strength and versatility of our foundation model, CT-CLIP. These results reaffirm the value of our approach and the generalizability of CT-CLIP's representations to a range of downstream tasks.

Comment 2: "CT foundational model" terminology overstates generalizability

Reviewer: The manuscript refers to a "CT foundational model," but it is trained solely on chest CT scans and validated on chest abnormalities. This raises a question about the broader applicability of the model. Given the ambitious terminology of "foundational," it would be helpful if the authors could either refine the objective to focus exclusively on chest CT or expand the model's validation to include other anatomical regions. If the latter approach is taken, this should be clearly stated in the manuscript to avoid ambiguity regarding the model's generalizability.

Response:
We thank the reviewer for pointing this out. Indeed, our model was specifically developed and validated on **chest CT** data, and the term "CT foundational model" may suggest a broader anatomical or modality-level generalization than is currently supported by our data. To address this, we have revised the manuscript to consistently use the term **"chest CT foundation model"** and clearly communicate the current scope of the model.

The limitation in scope is largely driven by the availability of data: to the best of our knowledge, **CT-RATE** remains the first and only publicly available dataset that pairs **volumetric 3D CT scans with full-text radiology reports**, making it uniquely suited for training vision-language models in this space. Our approach, while generalizable in principle, is currently bounded by the nature of available paired training data. We highlight this as both a limitation and an opportunity for the community.

Comment 3: Limited external validation

Reviewer: The validation is performed on a single external dataset. While this is useful, it limits the robustness of the conclusions drawn. I recommend that the authors expand their validation efforts by including the M3D dataset, which is more diverse and includes a broader range of cases. Additionally, it would be valuable to report the model's zero-shot performance on both common and rare diseases separately, which would offer a more nuanced understanding of the model's capabilities and limitations.

Response:
We fully agree that broader validation across multiple datasets would significantly strengthen the claims. Unfortunately, while the **M3D dataset** appears diverse, it primarily comprises 2D JPEG slices collected from Radiopaedia and does not provide sufficient and reasonable chest CT volumes suitable for our 3D models. Furthermore, many of the reported numbers in M3D refer to **slice counts**, not volume counts, making it unsuitable for volumetric evaluation.

As a stronger alternative, we followed up on the reviewer's earlier mention of **MedSyn**, which uses an in-house dataset combining 3D CTs and reports. We contacted the MedSyn authors, and they graciously agreed to evaluate our model on their dataset. The results of this **external validation** are now included in **Figure 4a**, with additional details in the *"Adding external validation datasets to CT-RATE."* of the Methods section.

Regarding the evaluation of performance across **common vs. rare diseases**, we have now included a deeper analysis and discussion. While **Figure 2b** presents the abnormality distribution and Extended Data Figures 3 and 4 report class-wise results, we agree that these findings were not previously discussed in sufficient depth. Upon further analysis, we noticed that performance discrepancies are **not primarily driven by frequency**, but instead by **abnormality size**. For example, large-scale abnormalities like cardiomegaly are consistently better detected than smaller ones like pulmonary nodules—regardless of prevalence. This observation suggests future work should prioritize improving small-lesion detection through better spatial encoding in the vision backbone.

Comment 4: Manuscript would benefit from ablation studies on model design

Reviewer: The manuscript would benefit from a thorough ablation study to highlight the contributions of various design choices. For example, the impact of including different sections of the reports, the effect of volume resizing, and the comparative performance of vocabfine vs classfine should all be systematically analyzed. Such an ablation study would provide a deeper understanding of the model's strengths and limitations, as well as guidance for future improvements.

Response:
We thank the reviewer for this insightful and important comment. We fully agree that systematic ablation studies are critical to understanding the contributions of individual components and design choices within a complex model. While we have already conducted a broad range of ablation experiments, we acknowledge that many of them are placed in the supplementary materials and may not have been sufficiently highlighted or discussed in the main text. We have now revised the manuscript to ensure that these evaluations and their implications are more clearly articulated.

For instance, to assess the impact of report content on model learning, we performed an ablation study examining how different sections of the radiology reports—such as the findings, impression, or combinations thereof—affect model performance. These results are included in **Supplementary Figure 1a** and are now discussed in the *"CT-CLIP: Developing a visual-language foundation model with CT-RATE"* section of the Results. We also conducted a thorough comparison between the two finetuning strategies introduced in the paper: VocabFine and ClassFine. This comparison is illustrated in **Figure 4a**, **Extended Data Figures 3 and 4**, and **Supplementary Figures 5 and 9**. In response to both this comment and similar concerns raised by Reviewer #2, we elaborated it in the *"Enhancing CT-CLIP's detection performance with finetuning"* of the Results section and in the Discussion section to clarify the trade-offs between these methods. In summary, VocabFine enables flexible cross-modal tasks such as report-to-volume retrieval by fine-tuning both encoders, while ClassFine is more lightweight and efficient for vision-only tasks.

Beyond these, we also evaluated several other important design factors. In **Figure 4b**, we analyzed seven different prompt formulations, commonly found in our report corpus, to identify optimal prompt engineering strategies for zero-shot classification. We further explored how CT-CLIP performance scales with varying amounts of training data by training the model with 10%, 20%, 40%, 60%, 80%, and 100% of CT-RATE (**Figure 4c**), providing insights into data efficiency. For CT-CHAT, we carried out an ablation study on the large language model backbone (**Figure 6d**) to identify which pretrained LLM integrates best with our 3D vision encoder (pretrained via CT-CLIP). **Supplementary Table 10a** evaluates the necessity of 3D volume input for CT-CHAT, demonstrating that the model's answers are grounded in actual imaging content. Additionally, **Supplementary Table 10b** presents an ablation study on the effect of concept alignment pretraining, revealing that its contribution to final performance is limited. Finally, we analyzed different finetuning strategies in **Supplementary Figure 1b**, comparing fully frozen vision encoders to partially updated models for both VocabFine and ClassFine, which guided our final training choices. We believe these extensive ablations provide a comprehensive understanding of the model's strengths and limitations. We have now improved the visibility and discussion of these results in the main text, and we are grateful to the reviewer for encouraging us to more clearly present and interpret these critical analyses.

Rebuttal to Reviewer #2

Comment 1: Clarify trade-offs between ClassFine and VocabFine

Reviewer: The manuscript could detail the trade-offs between ClassFine and VocabFine approaches to better inform their application in specific use cases. Such as the reason why these finetuning methods are used to evaluate retrieval tasks are not clear.

Response:
We thank the reviewer for highlighting this important point. Indeed, we performed detailed comparisons between **ClassFine** and **VocabFine**—see **Figure 4a**, Extended Data Figures 3 and 4, and Supplementary Figures 5 and 9. However, as the reviewer rightly noted, the **rationale and trade-offs** between these two approaches were not clearly emphasized in the main text. We now elaborate these distinctions in the revised *"Enhancing CT-CLIP's detection performance with finetuning"* of the Results section and in the Discussion Section.

ClassFine adds a lightweight classification head on top of the frozen vision encoder, making it suitable for **vision-only tasks** like multi-label classification or volume-to-volume retrieval. And **VocabFine** fine-tunes both the vision and text encoders with contrastive objectives, enabling **cross-modal tasks** such as **report-to-volume retrieval**. Thus, ClassFine is more lightweight but less flexible, whereas VocabFine enables broader use cases and better cross-modal generalization. Regarding the reviewer's question on why we evaluate these fine-tuned models on retrieval tasks: our goal was to investigate how these fine-tuning strategies affect the representational capacity of the CT-CLIP encoders. Interestingly, we observe that all models exhibit similar performance, with one notable exception—VocabFine shows reduced performance in report-to-volume retrieval. This suggests that the VocabFine fine-tuning may have significantly altered the text encoder weights, while leaving the vision encoder largely unaffected. Overall, this supports the observation that ClassFine maintains a more stable representation in the frozen encoder, while VocabFine introduces broader changes that benefit cross-modal learning but may affect alignment in certain tasks. We have clarified these insights and trade-offs more explicitly in the revised Results and Discussion sections, and we hope this addresses the reviewer's concern regarding the purpose and interpretation of these evaluations.

Comment 2: Include detection/segmentation tasks for practical relevance

Reviewer: The inclusion of experiments related to detection and segmentation tasks, which are critical in CT imaging, would enhance the study's practical relevance.

Response:
We fully agree with the reviewer on the clinical importance of segmentation and localization tasks in 3D CT imaging. Unfortunately, to the best of our knowledge, no **publicly available 3D chest CT dataset** exists that provides **multi-abnormality segmentation masks**, which severely limits opportunities to evaluate this aspect directly. Additionally, CT-RATE does not currently include expert-generated masks. That said, to move toward this goal, we have integrated **organ-level anatomical segmentations** using a state-of-the-art segmentation model. These are now **publicly available** through the CT-RATE release on Hugging Face and

can serve as a basis for visual grounding, anatomical localization, and future fine-grained abnormality segmentation. We have added this information in the *"Creating the CT-RATE dataset"* section of Method (see also Supplementay Table 11 for more details). As for enhancing CT-RATE with multi-abnormality segmentation masks, we recognize that this would require substantial expert annotation effort. We consider this a valuable direction and plan to pursue it as part of future work.

Comment 3: Show fine-tuning efficiency with smaller data fractions

Reviewer: As CLIP and VLM models aim to extract rich representations independent of predefined supervised classes, a demonstration of fine-tuning with a smaller portion of the data could provide valuable insights into the model's efficiency and adaptability.

Response:
We fully agree that evaluating the model's performance when fine-tuned on a smaller portion of the dataset would offer valuable insight into its efficiency and adaptability. However, we faced practical limitations in exploring this direction due to the scarcity of suitable public datasets. Among the few datasets available, RAD-ChestCT and UPMC (used in the MedSyn study) are the only ones that are somewhat comparable to CT-RATE. RAD-ChestCT is already relatively small—containing less than 10% of the number of cases in CT-RATE—and does not include radiology reports, making it suitable only for classification-based external validation. UPMC, on the other hand, is a private dataset and not publicly accessible. We were able to collaborate with the MedSyn team to evaluate our models on their test set, but broader experiments involving varying training sizes were not feasible due to these access constraints.

Nevertheless, we believe our current experiments do offer some insights into the robustness and generalizability of CT-CLIP. Importantly, both the ClassFine and VocabFine models are trained solely on CT-RATE, yet they achieve strong performance on external datasets such as RAD-ChestCT and UPMC. This indicates that the contrastively pretrained CT-CLIP encoder learns highly transferable representations that generalize well under domain shift. These findings are discussed in the *"Enhancing CT-CLIP's detection performance with finetuning"* section of the Results.

Comment 4: Add clinically relevant metrics for report generation evaluation

Reviewer: For Figure 6, metrics such as BLEU and ROUGE are text-focused and may inherently favor the model due to differences in training modalities. Adding a clinical relevance metric—similar to an adaptation of ChexBert F1 for CXR—would provide a more reliable assessment of report informativeness. The authors can use their own trained report labeler model for this.

Response:
We completely agree with the reviewer, and we sincerely thank you for this valuable suggestion. As noted, standard natural language generation (NLG) metrics such as BLEU and ROUGE are limited in their ability to capture the clinical accuracy and informativeness of

generated responses. They often emphasize surface-level textual overlap, which may not correlate well with diagnostic correctness, particularly in cross-modal settings.

Motivated by this, we incorporated a clinically meaningful evaluation by leveraging our own trained automated report labeler—originally used in CT-RATE to annotate radiology reports (as suggested by the Reviewer, we appreciate the idea). This labeler allows us to assess the diagnostic precision, recall, and F1 score of the generated reports, offering a more clinically grounded measure of informativeness. The results are reported in **Figure 6e** and discussed in detail in the *"Surpassing other multimodal AI assistants with CT-CHAT"* section of Results.

To ensure a fair evaluation, rather than comparing CT-CHAT with 2D vision-language assistants—which are optimized for fundamentally different input domains—we benchmarked CT-CHAT's report generation against recent state-of-the-art 3D report generation models, namely **RadFM** and **CT2Rep**. Despite CT-CHAT being primarily trained for VQA rather than generation, it outperforms both baselines on clinical metrics. This is particularly notable given that CT2Rep is trained on the same CT-RATE dataset. However, we also acknowledge that while these results are promising, the quality of CT-CHAT's generated reports remains below the threshold needed for clinical deployment. We highlight this limitation in the manuscript and agree that future work should further refine both generation quality and clinical validation.

Once again, we appreciate the reviewer's suggestion, which helped us strengthen the clinical relevance and robustness of our evaluation.

Rebuttal to Reviewer #1

Comment 1: Recommendation for Independent Expert Evaluation

Reviewer: However, I believe it is essential to have a minimum of three clinical experts review the quality of the reports and chat outputs, specifically assessing comprehensiveness, hallucinations, and missing information, before the manuscript is considered for publication.

Response: We thank the reviewer for highlighting the importance of clinical expert evaluation for report generation and chat quality, we fully agree that expert assessment is essential to ensure comprehensiveness and minimize hallucinations in the generated reports by CT-CHAT.

- In response, we conducted detailed evaluations involving **three board-certified radiologists**, as described in the **"Surpassing other multimodal AI assistants with CT-CHAT"** section of **Results** (see *page 7* and *Figure 6d*).
- Each radiologist independently assessed 150 reports generated by CT-CHAT, CT2Rep, and RadFM, scoring them on clinical accuracy and completeness.
- The results demonstrate that *Radiologist-1 scored CT-CHAT at 3.44**,** Radiologist-2 at 4.10**,*** and *Radiologist-3 at 4.52**,*** all consistently higher than competing methods. This indicates clear expert approval of CT-CHAT's superior performance while also recognizing areas for many further improvement before clinical deployment.
- These results are visualized in **Figure 6d** and discussed in detail in the **Results**, **Discussion** and **Online Methods** sections.

We believe these expert evaluations provide a strong and transparent foundation for assessing report quality. Furthermore, by releasing CT-CHAT and CT-CLIP, together with the CT-RATE dataset, as open-source resources, we aim to lay important groundwork for future development and refinement toward the ultimate clinical deployment of 3D CT AI assistants.

Rebuttal to Reviewer #2

Comment 1: Suggestion to Broaden Downstream Task Evaluation

Reviewer: The absence of a wider variety of downstream tasks (such as segmentation and detection) limits the demonstration of the model's representational power. However, this is understandable given the current lack of public data with such annotations. Could the authors consider adapting a downstream task using LUNA16 (for nodule detection), to further demonstrate model versatility?

Response: We thank the reviewer for this comment and agree that demonstrating performance across diverse downstream tasks would further highlight the representational power of foundation models. However, our core goal with **CT-CLIP** and **CT-CHAT** is to learn *generalizable representations* for 3D chest CTs, leveraging the broad spectrum of findings in

our dataset. The reports in **CT-RATE** cover all chest-related findings, including over **100 abnormality types**, spanning both common and rare conditions. In contrast, specialized datasets like **LUNA** focus mainly on lung nodules, making them valuable but narrow in scope. Competing directly with specialized models on highly localized tasks like nodule detection would therefore be challenging for our generalist models and beyond our current scope.

Inspired by the reviewer's suggestion, however, we explored how specialized detection models can enhance generalist pipelines. Specifically, we utilized a state-of-the-art nodule segmentation algorithm trained on **LUNA16** to generate nodule masks for the CT-RATE dataset. This information was then used to refine the **CT-CHAT** report generation pipeline, as detailed in the **"Developing the CT-CHAT model"** section of the **Online Methods**.

As shown in **Figure 6e**, the addition of structured 3D nodule masks boosted clinical accuracy not only for nodules but also for most other pathologies, with the mean F1 score improving by approximately 70% compared to the base model. While we currently lack large-scale, high-quality 3D segmentation models for other pathologies, mainly due to the limited availability of suitable annotated datasets, this experiment demonstrates that incorporating robust segmentation modules is a promising direction for bringing generalist vision-language models closer to clinical deployment. Resources like **CT-RATE**, with its breadth of paired 3D imaging and reports, can help accelerate the development of such specialized models in the future.

In summary, our CT-RATE dataset, CT-CLIP, and CT-CHAT models aim to lay the groundwork for generalist 3D chest CT AI. We appreciate the reviewer's comment, which inspired us to explore this direction, and we plan to extend this line of work by integrating more specialized modules as segmentation models for other pathologies become available

---

Minor Comment 1: Clarification on Drafting and Translation

Reviewer: For transparency and scientific integrity, I recommend that the authors clarify whether the manuscript was originally drafted in English or translated from another language. If translation was involved at any stage, please provide details on the process and whether professional translation or native English editing was used.

Response: We confirm that the manuscript was **originally drafted in English**. We used a large language model for minor editing and grammar checks to improve readability. However, we acknowledge that some sections of the text may still have flow issues, and in this revision, we have carefully addressed these points to ensure a clearer structure and smoother phrasing.

Minor Comment 2: Concern About Clinical Readiness of Report Generation

Reviewer: While it's good to see clinical F1, precision, and recall metrics included for report generation, the actual F1 scores are still quite low. This shows that, even though there's been improvement compared to previous models, the report generation is still far from being ready for clinical use. I suggest that the authors talk more about this limitation in the paper and share some ideas or plans for how this could be improved in the future.

Response: We thank the reviewer for highlighting this important point. We definetely agree that, despite substantial progress, the current performance remains insufficient for real-world clinical deployment of report generation in 3D medical imaging. Although we discussed this limitation in the Discussion section, we recognize that it warrants greater emphasis. In this revision, we have expanded this discussion to clearly acknowledge that significant further research and development will be required before clinical adoption is feasible, and we have outlined our perspective on how to move toward that ultimate goal. We have added more details about potential future directions, including the integration of high-quality structured segmentation and detection modules, improved multimodal alignment strategies, and the expansion of diverse, structured, high-quality datasets. While our open-source resources (CT-RATE, CT-CLIP, and CT-CHAT) provide a strong foundation, we acknowledge that they represent only initial steps toward this long-term objective.

Optional Suggestions: Comment on Dataset Size and Accessibility

Reviewer: The open-access dataset's image files are very large. Providing a version with reduced image size or compressed data types would be extremely helpful to many researchers and could increase accessibility.

Response: We thank the reviewer for this practical and valuable suggestion. In response, we have released a lighter and enhanced version of the CT-RATE dataset on our Hugging Face repository. In this version, the 3D CT scans are stored as int16 instead of float32, which significantly reduces the overall file size without compromising essential Hounsfield unit information. Additionally, we have fixed and standardized the NIfTI header information, including slice spacing and thickness, to improve usability across common medical imaging tools. We hope this more accessible version will better support the community.

Remarks on Code Availability

Reviewer: Although there are some minor issues in the code, they do not prevent its use, and it should be straightforward for others to retrain the models if needed.

Response: We thank the reviewer for acknowledging the reproducibility of our codebase and for highlighting the minor issues. We carefully reviewed the entire pipeline, resolved the issues, and rerun all steps to ensure everything works smoothly. We have also updated our GitHub repositories to address these points and further support reproducibility within the community.
